# Tough Materials Through Ionic Interactions

**DOI:** 10.3389/fchem.2021.721656

**Published:** 2021-07-27

**Authors:** Linda Salminen, Erno Karjalainen, Vladimir Aseyev, Heikki Tenhu

**Affiliations:** ^1^ Department of Chemistry, University of Helsinki, Helsinki, Finland; ^2^ VTT Technical Research Centre of Finland Ltd., Espoo, Finland

**Keywords:** photopolymerization, dynamic crosslinker, crosslinking, reinforcement, tensile strength

## Abstract

This article introduces butyl acrylate-based materials that are toughened with dynamic crosslinkers. These dynamic crosslinkers are salts where both the anion and cation polymerize. The ion pairs between the polymerized anions and cations form dynamic crosslinks that break and reform under deformation. Chemical crosslinker was used to bring shape stability. The extent of dynamic and chemical crosslinking was related to the mechanical and thermal properties of the materials. Furthermore, the dependence of the material properties on different dynamic crosslinkers—tributyl-(4-vinylbenzyl)ammonium sulfopropyl acrylate (C4ASA) and trihexyl-(4-vinylbenzyl)ammonium sulfopropyl acrylate (C6ASA)—was studied. The materials’ mechanical and thermal properties were characterized by means of tensile tests, dynamic mechanical analysis, differential scanning calorimetry, and thermogravimetric analysis. The dynamic crosslinks strengthened the materials considerably. Chemical crosslinks decreased the elasticity of the materials but did not significantly affect their strength. Comparison of the two ionic crosslinkers revealed that changing the crosslinker from C4ASA to C6ASA results in more elastic, but slightly weaker materials. In conclusion, dynamic crosslinks provide substantial enhancement of mechanical properties of the materials. This is a unique approach that is utilizable for a wide variety of polymer materials.

## Introduction

Additive manufacturing (AM) has emerged an increased demand of new, rapidly photopolymerizable materials. Photopolymerization, which is also known as photocuring or photo-crosslinking, is a technique with which liquid-state monomers or oligomers are polymerized upon exposure to light. When exposed to light, a photoinitiator forms reactive species such as radicals, which initiate polymerization (Bagheri and Jin, 2019). A commonly found drawback of radical cured photopolymers is their insufficient toughness (Oesterreicher et al., 2016). Hence, new methodologies are needed to improve them.

Polymer networks are commonly classified as either physical or chemical networks. The chains of a chemical network are connected by covalent bonds, whereas those of a physical network are connected through non-covalent interactions (Seiffert and Sprakel, 2012). Classically elastomers have been crosslinked via permanent covalent bonding, while ionic or electrostatic interactions provide an alternative way to crosslink polymers (Tant and Wilkes, 1988). Ionomers, polymers that contain small percentages of ionic repeat units, have been described as self-assembled composites in which ions form physical aggregates that serve as crosslinks (Ruan and Simmons, 2015).

Ionomers are of interest, since the inclusion of ions into organic polymers affects their physical properties (Hara and Jar, 1988; Eisenberg et al., 1990; Visser and Cooper, 1991). Inclusion of a small amount of charged groups alters the glass transition temperature and segmental dynamics of the polymer (Gauthier and Eisenberg, 1990). The ion pairs in random ionomers form two types of aggregates—multiplets and clusters—according to the ion content (Hird and Eisenberg, 1990). At low ion contents, small ionic aggregates that are known as multiplets dominate (Hird and Eisenberg, 1990). Multiplets act as crosslinkers and increase the modulus and glass-transition temperature (*T*_g_) of the polymer (Hird and Eisenberg, 1992). The ion pairs of the multiplets yield regions of restricted mobility by anchoring the polymer chains near the multiplets (Eisenberg et al., 1990). Above a threshold ion content, clusters are the dominating aggregate-type (Hird and Eisenberg, 1990). They are aggregates that consist of ion pairs and portions of hydrocarbon chains of the matrix. Clusters form a second phase that exhibits its own glass transition temperature and acts like a reinforcing filler (Bellinger et al., 1994).

Not only have the ion-aggregates altered the glass transition temperatures of organic polymers, but they have also led to improved mechanical properties (Hara et al., 1988; Hara and Jar, 1988; Bellinger et al., 1994; Lee et al., 1998). For instance, the tensile strength and toughness of sulfonated polystyrene ionomers has had significant enhancements with the inclusion of ionic moieties (Bellinger et al., 1994). Deformation and fracture studies of the polymers exhibited changes in their fatigue performance with varying ion content (Hara and Jar, 1988). The deformability of the ionomer changed as the ion content surpassed a threshold level (Hara and Jar, 1988). At low ion contents, the multiplets formed crosslinks that made the ionomer brittle, whereas above the threshold, the fatigue performance improved with increasing ion content (Hara et al., 1988). The enhancements were attributed to ionic clusters that functioned as reinforcing fillers, and provided effective ionic crosslinks (Hara et al., 1988; Hara and Jar, 1988; Bellinger et al., 1994).

Photopolymerized materials are widely used but suffer from lacking material properties. Sacrificial bonds – weak bonds that break in favor of stronger bonds—offer a mechanism for toughening polymers. Energy dissipation by weak reversible bonds, such as ionic interactions, serves to preserve the more strong, covalently bonded network (Luo et al., 2015; Neal et al., 2015; Yoshida et al., 2017).

This contribution introduces a new, widely applicable approach for preparing photopolymers with excellent mechanical properties. This is realized by crosslinking traditional non-charged monomers with a chemical crosslinker and a dynamic crosslinker: a salt that contains polymerizable double bonds in both its anion and cation. Chemical crosslinker stabilizes the form and the ion pairs in the dynamic crosslinker act as sacrificial bonds that can break and reform under stress (Zhou et al., 2017). Utilization of both types of crosslinks results in tough materials that can withstand high stress and deformation. The mechanical and thermal properties of such materials were studied with dynamic mechanical analysis (DMA), differential scanning calorimetry (DSC), thermogravimetric analysis (TGA), and stress-strain measurements.

## Materials and Methods

### Materials

Butyl acrylate (BuA) (Aldrich, 99%) was distilled and stored in a freezer. 1,4-Butanediol dimethacrylate (BudMA) (Aldrich, 95%) was passed through basic Al_2_O_3_ and filtered. 2,2′-Azobis (2-methylpropionitrile) (AIBN) (Fluka, 98%), was recrystallized from methanol. 2,6-Di-tert-butyl-4-methylphenol (BHT), 3-sulfopropyl acrylate potassium salt (SAK) (Aldrich), 2-hydroxy-2-methylpropiophenone (HMPP) (Aldrich, 97%), and 2-cyano-2-propyl benzodithioate (2-C2PBDA) (Aldrich, 97%) were used as received.

### Syntheses

***Tributyl-*** (***4-vinylbenzyl***) ***ammonium chloride*** (***C4ACl***) ***.*** 4-Vinylbenzyl chloride (10.0028 g, 65.5 mmol), tributylamine (13.3650 g, 72.1 mmol), and BHT (0.0662 g) were dissolved in 20 ml of acetone. The mixture was let to react at 50°C for 24 h. The product was isolated by precipitating it twice in cold diethyl ether. The product was dried under vacuum (Yield 7.9709 g, 35.99%).

***Trihexyl-*** (***4-vinylbenzyl***) ***ammonium chloride*** (***C6ACl***) ***.*** 4-Vinylbenzyl chloride (5.0063 g, 32.8 mmol), trihexylamine (9.7134 g, 36.0 mmol), BHT (0.0985 g) were dissolved in 10 ml of acetone. The mixture was let to react at 50°C for 64 h. The product was purified by precipitating twice in cold diethyl ether (Yield 7.2883 g, 52.64%).

***Tributyl-*** (***4-vinylbenzyl***) ***ammonium sulfopropyl acrylate*** (***C4ASA***) ***.*** C4ACl (6.6054 g, 19.5 mmol), SAK (4.9908 g, 21.5 mmol), and BHT (0.0347 g) were mixed in 50 ml of acetonitrile and stirred at room temperature for 70 h. The solids were removed through filtration, and the filtrate was eluted with acetonitrile through a short silica column. The solution was filtrated and acetonitrile was evaporated. The remaining ionic liquid was dried under vacuum (Yield 9.1590 g, 94.5%).

***Trihexyl-*** (***4-vinylbenzyl***) ***ammonium sulfopropyl acrylate*** (***C6ASA***) ***.*** C6ACl (5.0070 g, 11.9 mmol), SAK (3.0316 g, 13.1 mmol), and BHT (0.0266 g) were mixed with 50 ml of acetonitrile and stirred for 40 h. The solids were filtered off and the filtrate was eluted through a short silica column with a large amount of acetonitrile. After rinsing, the solution was filtrated, after which the solvent was evaporated. The product was dried under vacuum (Yield 6.6248 g, 96.3%).

***Poly*** [***trihexyl-*** (***4-vinylbenzyl***) ***ammonium chloride***] (***PC6ACl***) ***.*** C6ACl was polymerized via reversible addition fragmentation polymerization (RAFT). C6ACl (14.0011 g, 33.2 mmol), 2-C2PBDA (0.0736 g, 0.33 mmol), and AIBN (0.0056 g, 0.034 mmol) were dissolved in 50 ml dimethylformamide. The reaction mixture was purged with nitrogen for an hour, and the mixture was let to react at 100°C for 66 h. The polymer was purified by dialysing against water for 11 days, changing the water twice a day. The polymer was isolated by freeze-drying (Yield 8.6398 g).

***Poly*** [***trihexyl-*** (***4-vinylbenzyl***) ***ammonium sulfopropyl acrylate***] (***PC6ASA***) ***.*** PC6ACl (8.5077 g, 20.2 mmol of repeating units), SAK (5.1539 g, 22.2 mmol), and BHT (0.0423 g) were mixed in 200 ml of acetonitrile and stirred for 43 h at room temperature. The mixture was filtered, and the solution was evaporated until dry. The remains were dissolved in chloroform and precipitated in cold hexane. 9.4356 g of anion-exchanged polymer was obtained.

### Film Preparation

Butyl acrylate films were prepared with varying ions, ion contents, and concentrations of chemical crosslinker. The molar concentration of ionic monomer was varied from 0 to 30%. The concentration of covalent crosslinker BudMA was 1, 2, or 5 M %. The amount of initiator was always 2% of butyl acrylate. The initiator is not considered for the molar percentages; the amount was measured with respect to butyl acrylate only. For example, a film with 30% C4ASA and 1% BudMA contains 69% butyl acrylate.

The films were always prepared in the same way: butyl acrylate, crosslinker, UV initiator, and ionic monomer – or in one case, a polymer – were mixed into an even mixture. 4.5 ml of the mixture was spread on a mold and pressed even with a glass lid. The mixture was then polymerised by exposing it to UV light for 90 min. It should be noted that borosilicate is relatively transparent at 365 nm, which was the wavelength used in this work (Ehrt, 2002; Fujita et al., 2008). After polymerization the films were washed with acetonitrile, and dried. The purity of the films was verified with FTIR spectroscopy.

**FIGURE 1:**
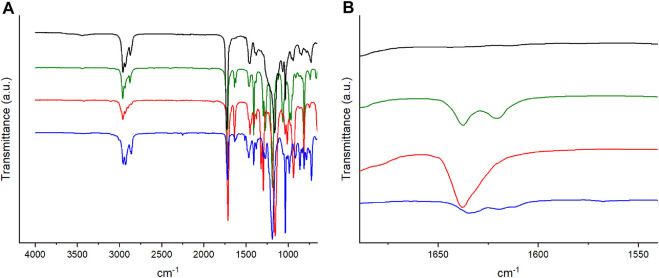
**(A)** The IR spectra and **(B)** a closeup of the IR spectra of a film with 2% BudMA and 5% C6ASA (black), butyl acrylate (green), BudMA (red), and C6ASA (blue). The spectra were shifted along the *y*-axis to facilitate comparison.

For example, a film with 2% BudMA and 5% C6ASA was prepared in the following way: BuA (4.0039 g, 31.2 mmol), BudMA (0.1516 g, 0.67 mmol), C6ASA (0.9756 g, 1.68 mmol), and 2-hydroxy-2-methylpropiophenone (0.1106 g, 0.67 mmol) were mixed into an even paste. Then 4.5 ml of the mixture was spread on a mold and polymerized by exposing it to UV light. After 1.5 h the film was removed from the mold and washed by soaking it in acetonitrile for one day, changing the acetonitrile twice during the process. The washed film was dried, and the absence of unreacted monomers was verified with IR spectroscopy. The verification was done by checking the absence of the C=C stretch band in the 1,600–1,650 cm^−1^ region (Figure 1).

**FIGURE 2:**
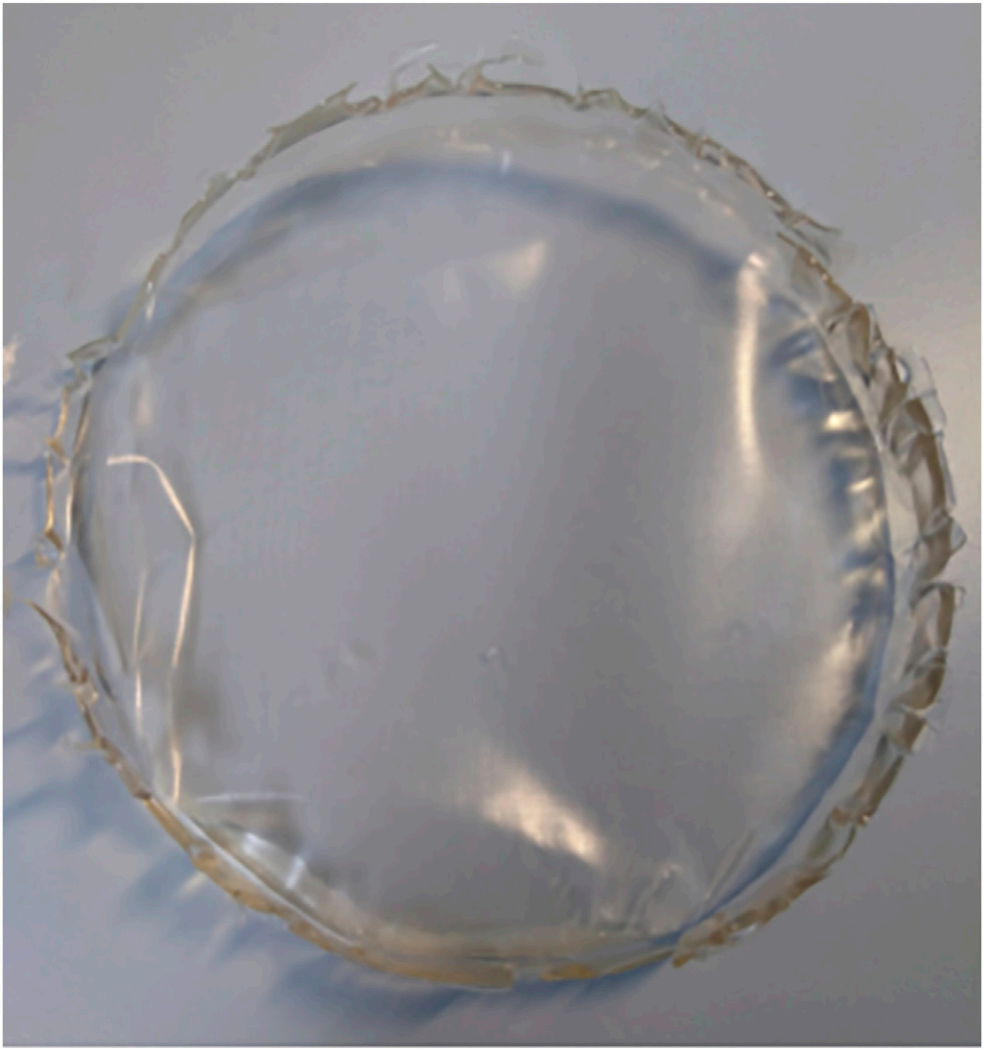
A film with 1% BudMA and 30% C4ASA.

To summarize, films with following compositions were prepared: 1) butyl acrylate, 1% BuA, and 0–30% C4ASA, 2) butyl acrylate, 2% BudMA, and 0–30% C4ASA, 3) butyl acrylate, 5% BudMA, and 0–30% C4ASA, 4) butyl acrylate, 2% BudMA, and 0–30% C6ASA, and 5) butyl acrylate, 2% BudMA, and 5% PC6ASA. The films were transparent and homogeneous, as is exemplified in Figure 2.

### Instrumentation

***Stress-strain measurements***. Stress-strain curves were measured with a Discovery HR-2 hybrid rheometer from TA Instruments. The films were stretched 2 μm/s until rupture. The environmental temperature was set to 25°C.

***Thermogravimetric measurements*** (***TGA***) ***.*** Thermogravimetric measurements were carried out with a Mettler Toledo TGA 850. The samples were heated from 25°C to 800°C at a rate of 10°C/min under nitrogen atmosphere.

***Oscillatory modulus measurements.*** The films’ moduli were measured with a DMAQ800 from TA Instruments. The moduli were measured from −50°C to 200°C with a heating rate of 5°C/min. The strain amplitude was 0.5% with frequency of 1 Hz.

***Differential scanning calorimetry*** (***DSC***) ***.*** Calorimetric measurements were conducted on a TA Instruments DSC Q2000 differential scanning calorimeter. The measurement programs were adjusted based on the decomposition temperature and expected *T*_g_ of the sample. Heating and cooling rates were always 20°C/min. Heating was started from −80°C.

## Results and Discussion

The following section describes the prepared materials. Then the results obtained from DMA, DSC, and stress-strain measurements will be presented.

Butyl acrylate was used as the matrix-forming monomer and is the major component in the studied systems. Acrylates are suitable for photopolymerization due to their fast radical chain growth that forms networks quickly (Gorsche et al., 2016). The glass transition temperature of butyl acrylate is low, −43 to −49°C, which helps the materials to retain their elasticity at room temperature (Penzel et al., 1997; White and Lipson, 2016). Also styrene – which is analogous to the cation – is polymerizable with UV light (Fujii, 1954; Zhang et al., 2007; Krüger et al., 2011). Covalent crosslinker (1,4-butanediol dimethacrylate, BudMA) was used to create chemical crosslinks that stabilized the produced films.

The dynamic crosslinkers, tributyl-(4-vinylbenzyl)ammonium sulfopropyl acrylate (C4ASA) and trihexyl-(4-vinylbenzyl)ammonium sulfopropyl acrylate (C6ASA) contain polymerizable double bonds both in their cations and anions. Therefore, the resulting polymer contains covalently bonded acrylate-based anions and styrene-based cations. Furthermore, the ionic interactions between the anions and cations form physical crosslinks that increase the stiffness of the materials. Although ion pairs have been used in the past to strengthen polymeric networks (Liu et al., 2010; Abouzari-Lotf et al., 2021), the utilization of salts where both the anion and cation polymerize is a novel approach.

Four different types of materials were prepared in the form of films. In the first film series, the concentration of covalent crosslinker was 1 mol%, and the concentration of ionic crosslinker, C4ASA, ranged from 0 to 30 mol%. Two additional sets of films were prepared with C4ASA as the ionic comonomer. These films were crosslinked with 2 mol% and 5 mol% of BudMA and the ion content was 0–30 mol%. The fourth type of material was prepared with a different dynamic crosslinker, trihexyl-(4-vinylbenzyl)ammonium sulfopropyl acrylate (C6ASA), and the molar concentration of chemical crosslinker was 2%. Again, the concentration of ionic crosslinker in the feed was varied from 0 to 30 mol%. All mentioned percentages are the molar concentration of the monomers in the feed. The films were thoroughly washed with acetonitrile and no unreacted monomer could be observed by IR spectroscopy (Figure 1). This indicates high purity of the synthesized films.

In addition to the four aforementioned film series, one film was prepared without chemical crosslinking. The film contained 30 mol% C4ASA, while butyl acrylate made up the remaining 70 mol%. This was done in order to study the effect of physical crosslinking in absence of chemical crosslinks. The film swelled in acetonitrile and formed a gel during the purification step of the synthesis. Thus, the sample was unsuitable for mechanical analysis. Hence chemical crosslinking was concluded to be necessary for the film synthesis with the current methodology.

**SCHEME 1:**
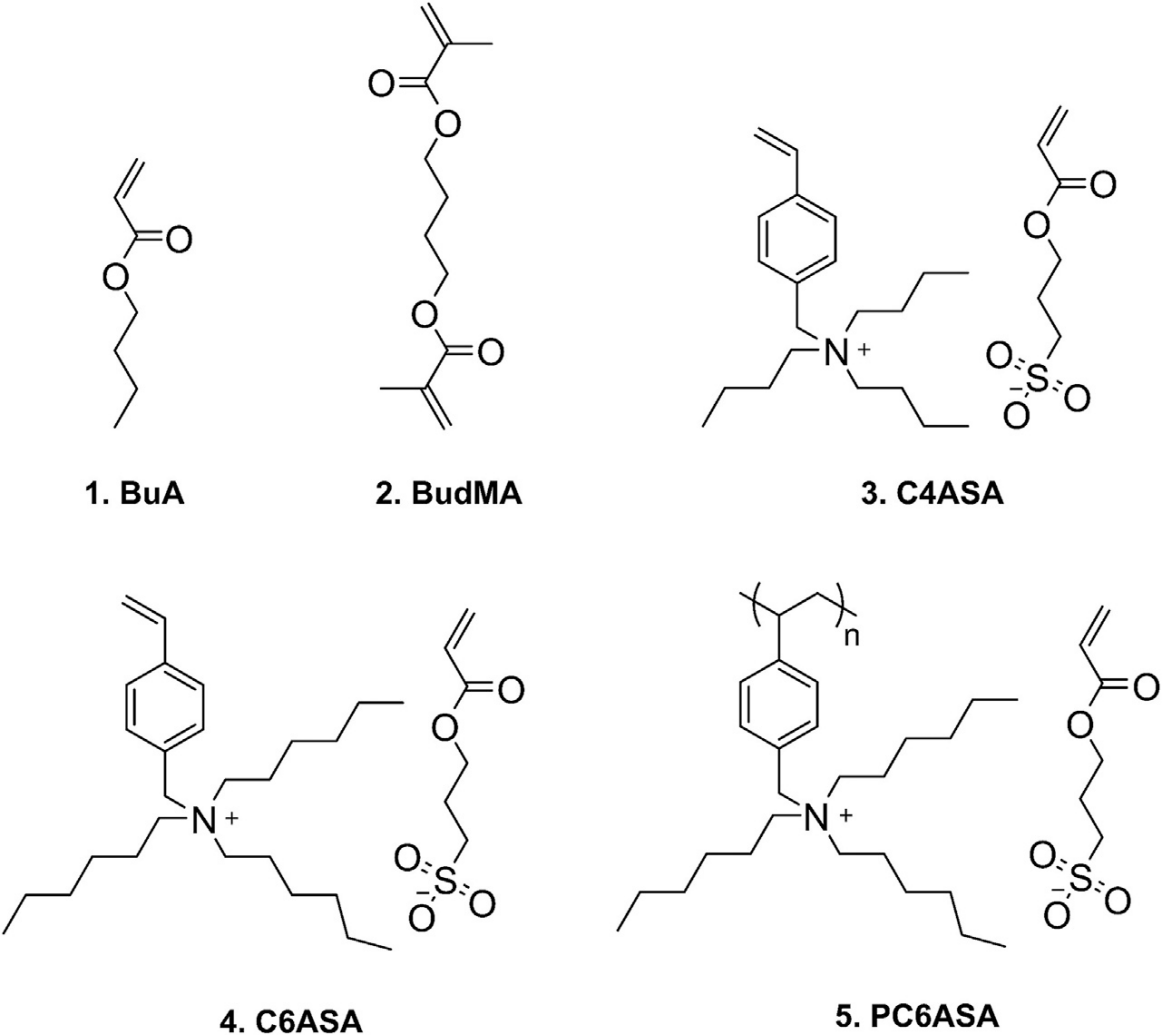
Structures of the monomers: 1. butyl acrylate (BuA), 2.1,4-butanediol dimethacrylate (BudMA), 3. tributyl-(4-vinylbenzyl)ammonium sulfopropyl acrylate (C4ASA), 4. trihexyl-(4-vinylbenzyl)ammonium sulfopropyl acrylate (C6ASA), and 5. poly [trihexyl-(4-vinylbenzyl)ammonium sulfopropyl acrylate] (PC6ASA).

Finally, a film was prepared with 5% of polymeric C6ASA (PC6ASA) in the feed. However, films with higher amounts of PC6ASA could not be prepared due to poor solubility of the polyelectrolyte in BuA. Scheme 1 illustrates the structures of butyl acrylate, the crosslinker, and the ionic monomers C4ASA and C6ASA, as well as PC6ASA. The NMR spectra of the dynamic crosslinkers are available as Supplementary Figures 1–3.

In short, the films can be divided into two classes: those that were prepared with 1) butyl acrylate, chemical crosslinker, and a salt with polymerizable anion and cation, and 2) the film that was prepared with butyl acrylate, chemical crosslinker, and polycation with polymerizable anion.

### Thermal Phase Transitions of C4ASA Films

The decomposition temperatures of the synthesized materials were determined through thermogravimetric analysis. For discussion on the thermal stability of the materials, see Supplementary Material. The decomposition temperatures were measured to determine a suitable temperature range for dynamic mechanical analysis (DMA). DMA was used to characterize the thermal phase transitions of the materials. The films’ storage and loss moduli were measured as a function of temperature, and their loss tangents (tan δ) were calculated. The amount of volatiles released from the films before decomposition is low (Supplementary Figures 4–7). This supports the results obtained from IR spectroscopy (Figure 1) that the films are essentially free of unreacted monomer.

**FIGURE 3:**
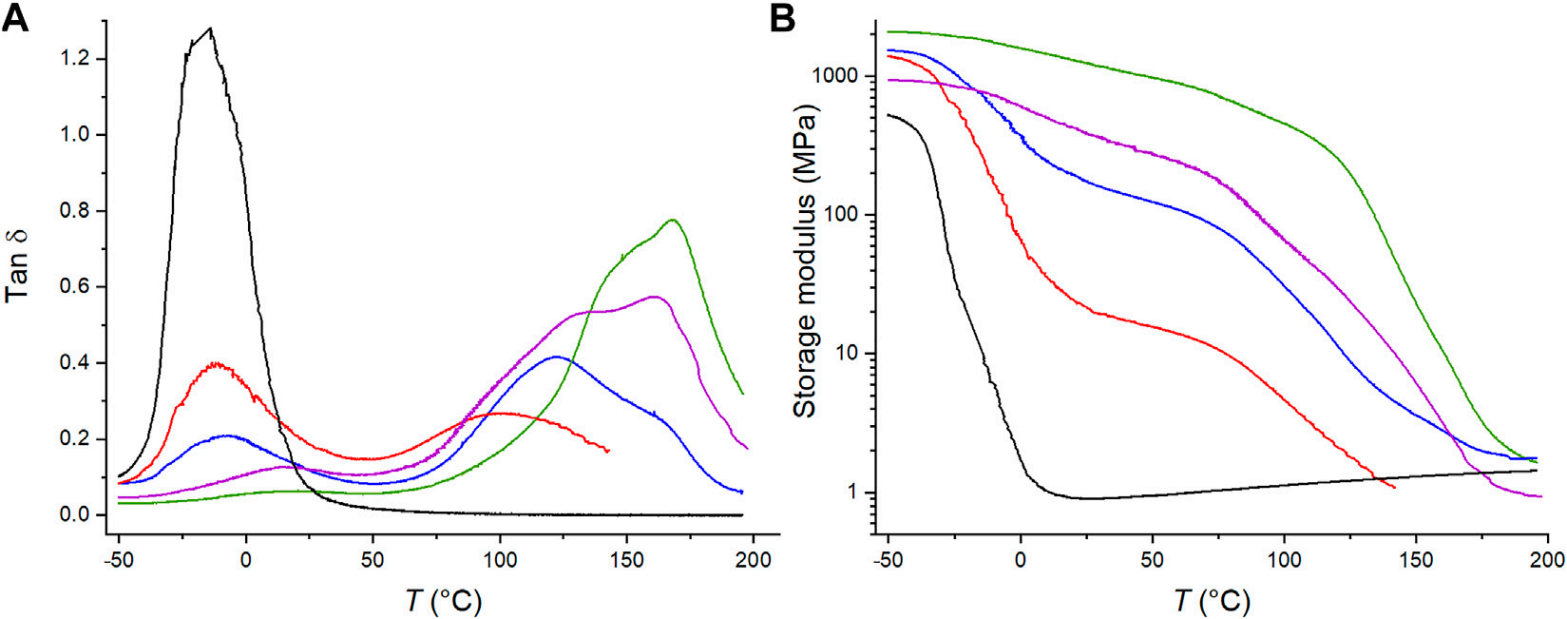
The temperature dependence of (A) the tan δ and (B) the storage modulus of BuA-films with 0% (black), 5% (red), 10% (blue), 20% (purple), and 30% (green) C4ASA. Each film contains 2% BudMA.

Figure 3A shows the tan δ curves of the C4ASA-films with 2% crosslinker as a function of temperature. The other films exhibit similar behavior and the obtained tan δ curves are given in Supplementary Material (Supplementary Figures 10–12). Copolymers comprising neutral and ionic monomers form phase separated ionic regions. The areas under the influence of the ionic regions have restricted mobility, and have higher glass transition temperatures than the surrounding matrix does (Eisenberg and Navratil, 1973; Hodge and Eisenberg, 1978).

Each film with an ionic comonomer exhibits at least two peaks. The first peak arises from the glass transition of butyl acrylate and the other from ionic cluster regions. The dimensions of the ionic regions must exceed a threshold value in order to exhibit a glass transition (Eisenberg et al., 1990). The lowest ion content, 5%, was already sufficient for exhibiting a cluster glass transition. Even with such a low ionic content, the height of the cluster peak is similar to the matrix peak, which suggests that even with the lowest ion content, an extensive region of restricted mobility is obtained. In a sense, the ionic regions can be seen as fillers in a “composite” in which the neutral polymer unaffected by the ionic regions forms the matrix. The areas of the matrix near the ionic moieties have restricted mobilities similarly to the filler-matrix interfaces in a composite. This then increases the *T*_g_ of the affected part of the matrix analogously to increase of *T*_g_ in a composite when fillers are introduced (Oh and Green, 2009; Qiao et al., 2011; Tessema et al., 2017). This effect is then observed as at least two glass transitions in the copolymer films. Some of the high-temperature transitions exhibit a shoulder in the high-temperature transition. This is attributed to heterogeneity of the materials. In particular, the distribution of BuA and crosslinks may vary, thus contributing to the observed heterogeneity. The peak broadness relates to the heterogeneity of the phase; broad peaks contain several regions of varying mobilities (Tsagaropoulos and Eisenberg, 1995). The broadest cluster peaks and shoulders are obtained with 10–20% ion content. When the ion content increases further, large continuous ionic regions form and the cluster peak narrows.

The storage modulus at the rubbery state is a measure for crosslink density and Figure 3B shows that the crosslink density increases with the ion content (Gorsche et al., 2016). Similar behaviour was observed for each material type – the storage modulus increases with increasing ion content. This is an indication of ionic crosslinking. Furthermore, Figure 3B shows that the films with 5, 10, and 20% C4ASA exhibit more than one transition, similarly to what was observed with the tan δ curves.

**FIGURE 4:**
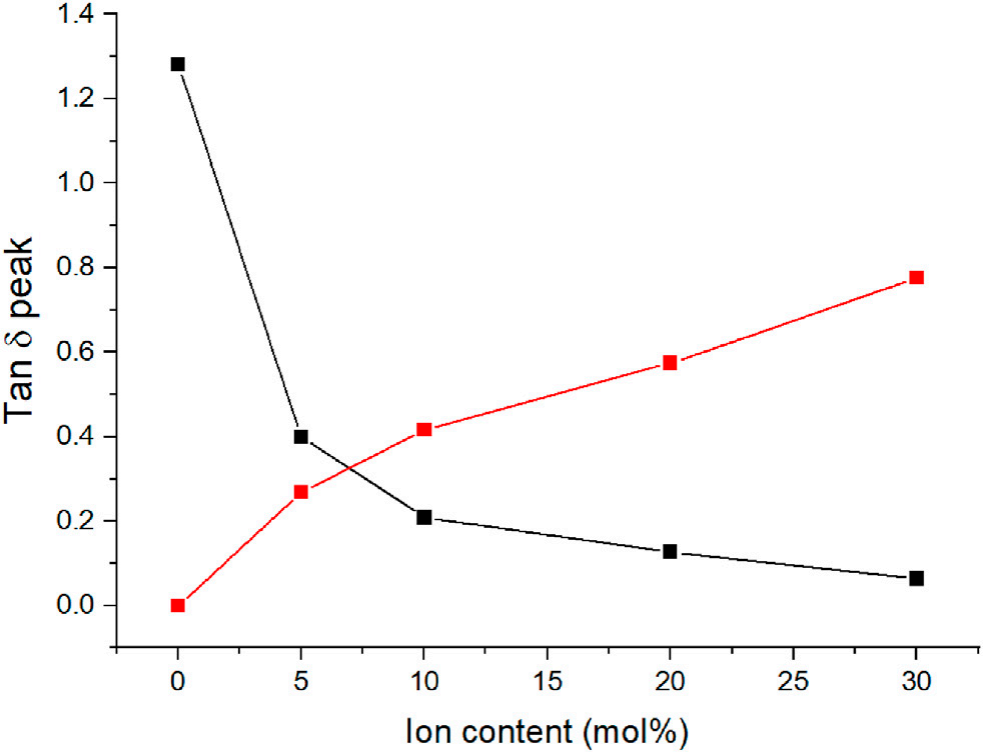
The tan δ peak heights of C4ASA-films as a function of ion content. The films are crosslinked with 2% BudMA. The matrix peak heights are given in black and the cluster peaks are given in red.

The height of the matrix *T*_g_ peak decreases in favor of the cluster *T*_g_ peak with increasing concentration of dynamic crosslinker. The intensity of the first peak decreases with increasing ion content and with the highest ion content studied, 30 mol%, the peak is barely discernible. With an ion content that high, the distances between ionic regions and the associated regions with restricted mobility overlap and large regions of restricted mobility form (Eisenberg et al., 1990). Figure 4 shows the tan δ peak heights as a function of ion content. The cluster phase becomes the dominant phase with approximately 7 mol% C4ASA. The other materials exhibit similar behavior; the cluster phase becomes dominant with slightly over 5 mol% of dynamic crosslinker in the feed (Supplementary Figures 13–15).

**FIGURE 5:**
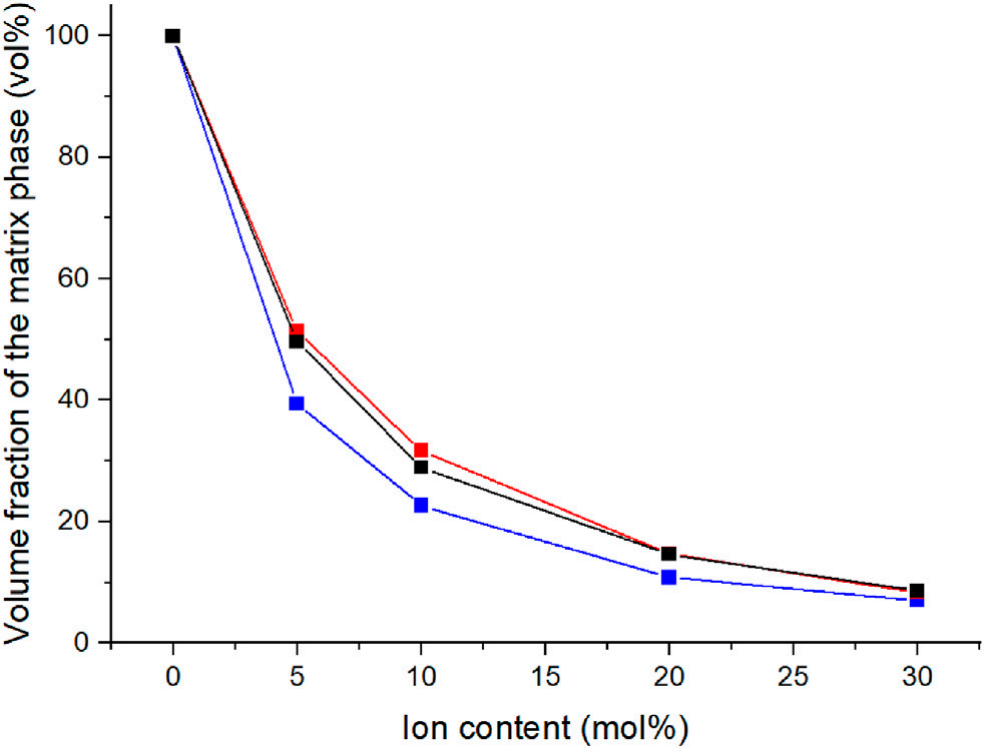
The ion content dependency of the volume fraction of the matrix phase. The volume fraction of the matrix phase was defined to be the area from under the lower temperature tan δ peak in relation to the area under entire tan δ curve. The materials have been crosslinked with 1% (blue), 2% (black), and 5% (red) BudMA.

The development of the volume fraction of the matrix phase can also be illustrated through the tan δ peak areas (Figure 5). The volume fraction of the matrix phase was determined from the areas under the tan δ peaks (Kim et al., 1994a). The volume fraction of matrix decreases with increasing ion content similarly to what was observed with the peak heights. Already with 5% C4ASA the volume fraction of the matrix phase is halved compared to the non-ionic material.

**FIGURE 6:**
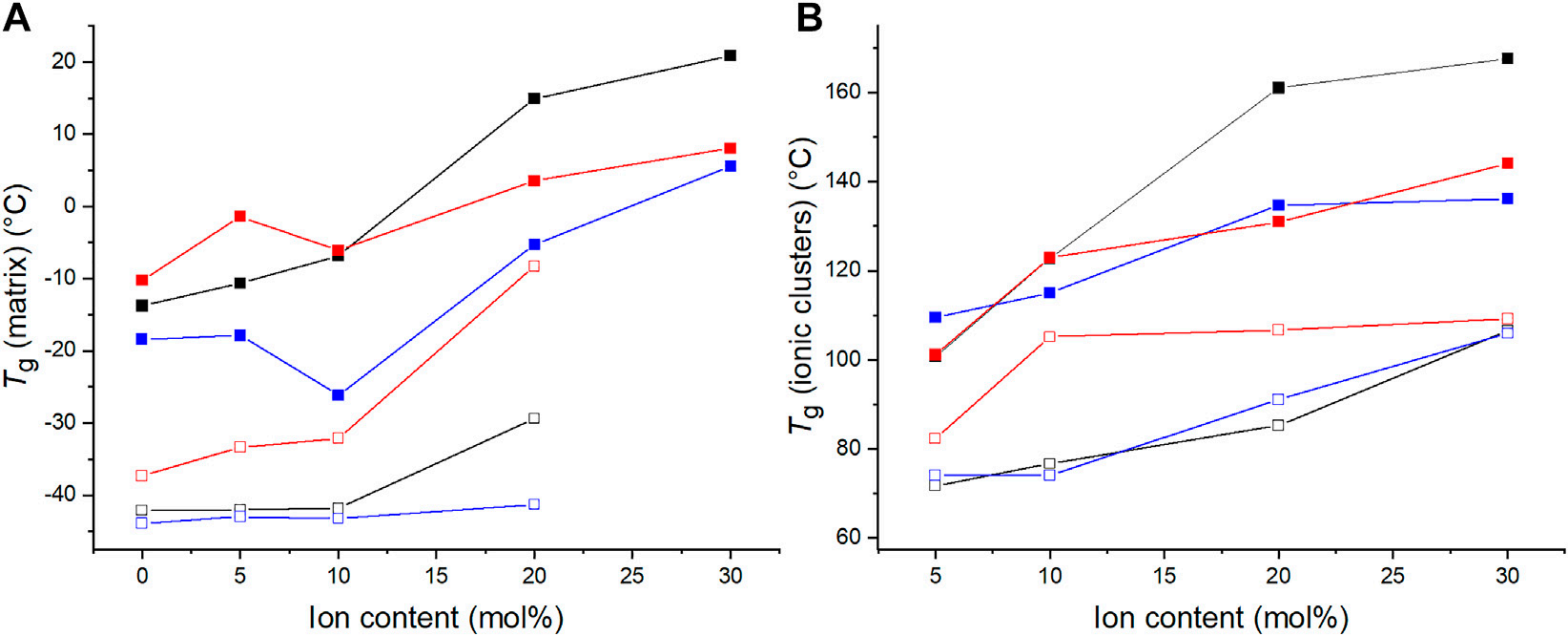
The glass transition temperatures of films with 1% (blue), 2% (black), and 5% (red) BudMA obtained with DMA (filled symbols) and DSC (empty symbols). The glass transition temperatures obtained with DMA (filled symbols) are defined as the peaks of tan δ curves. The *T*_g_ obtained by means of DSC (empty symbols) are the inflection points of the thermograms of the second heating run. **(A)** the *T*_g_ of the matrix phase and **(B)** the *T*_g_ of the regions of restricted mobility.

The materials’ *T*_g_ values were also determined by means of differential scanning calorimetry (DSC). The *T*_g_ values of the C4ASA-films are depicted below in Figure 6 together with the *T*_g_ values obtained with DMA. Both *T*_g_s increase with increasing ion content. The dynamic crosslinks restrict the chain movements, consequently delaying the glass transitions (Eisenberg et al., 1990). The increase of cluster *T*_g_ is an indication of cation-anion interactions and strengthened dipole-dipole interactions within ionic clusters (Wu and Weiss, 2004).

The films with different concentrations of chemical crosslinker differ slightly in their glass transition temperatures, although the differences do not relate directly to the concentration of BudMA. One might expect the *T*_g_ to increase with increasing concentration of crosslinker, considering that crosslinks generally increase the *T*_g_ of a polymer (Nielsen, 1969). Indeed, according to DMA the matrix *T*_g_ increases with increasing BudMA-concentration up until 10% ion content. However, thereafter the order changes and the materials with 2% BudMA exhibit the highest *T*_g_s. For example, the film that contains 2% BudMA and 20% C4ASA has a cluster *T*_g_ approximately 30°C above the *T*_g_ of a similar film with 5% BudMA. This implies that at least at high ion contents the reduced chain mobility from chemical crosslinks is not the main feature that determines the glass transition temperature. Instead, the significance of the ionic interactions prevails. The expected increase in *T*_g_ due to the formation of chemical crosslinks is compensated by reduction of *T*_g_ from weakened physical network. This means that although increasing the concentration of BudMA from 2 to 5% increases the density of chemical crosslinks, it disrupts the network of physical crosslinks, hence lowering the *T*_g_ of the material. DSC shows that the *T*_g_ increases with increasing BudMA concentration at all ion contents. DMA and DSC probe different phenomena and thus give different insights into the thermal behavior of the materials. DMA monitors the materials’ response to deformation, while DSC monitors changes in heat flow. Therefore, only DMA is able to detect the situation where increasing crosslink content disrupts the physical network.

The crosslinker is a type of a copolymerizing unit, whose addition changes the chemical composition of the material (Nielsen, 1969). Even though crosslinks themselves increase *T*_g_, depending on the nature of the crosslinker, the comonomer may either increase or decrease the *T*_g_ (Nielsen, 1969). The studied materials contain only 1–5% of crosslinker, which means that the effect change in monomer content is not a major factor in the observed shift in *T*_g_.

The *T*_g_ determined with DMA are larger than the *T*_g_s determined with DSC. *T*_g_ can be defined through several methods from a DMA measurement: the peak of tan δ curve, the onset of the drop in storage modulus, or the peak of loss modulus. The onset of storage modulus’ decrease and the peak of loss modulus are typically near the beginning of the transition, while the peak of tan δ is near the end of the transition (Achorn and Ferrillo, 1994). Since the glass transition occurs often on a wide temperature-range, the *T*_g_ can differ largely depending on the used definition (Hagen et al., 1994). To illustrate these differences, the DSC thermogram of a film with 2% BudMA and 5% C4ASA has been presented in Supplementary Figure 16 with the DMA data of the same material. The onset of the drop in storage modulus is in better agreement with the DSC *T*_g_, but the onset temperature is difficult to define precisely. The peak of loss modulus, which is also in a good agreement with DSC *T*_g_, offers an alternative method for evaluating *T*_g_. However, in the present case the high-temperature transition was not always definable in the loss modulus curves. Therefore, in this work the peak of tan δ was used as the definition of DMA *T*_g_ even though the values are higher than DSC *T*_g_. Even though the two measurement techniques have their differences and yield different *T*_g_ values, the trend of increased *T*_g_ with increasing degree of ion content is seen with both measurement methods.

**FIGURE 7:**
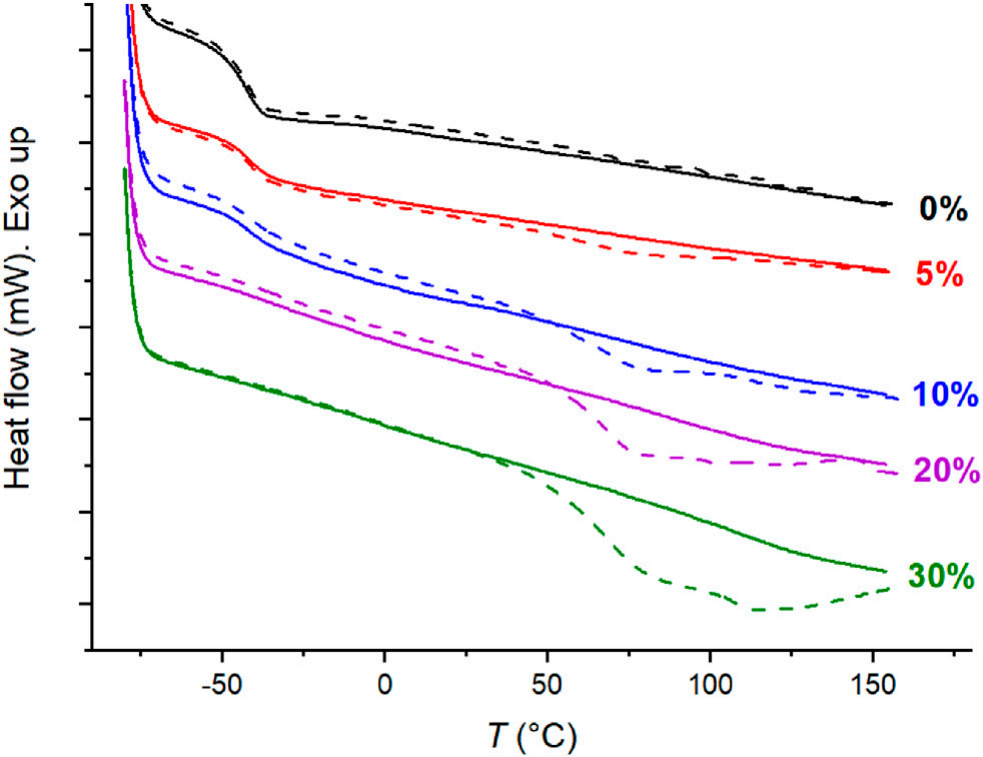
The DSC traces of films with 2% BudMA and varying concentrations of C4ASA. The first heating runs are depicted with dashed lines and the second heating runs with solid lines. The sample was stabilized for 5 min at -80°C before the second heating run. The DSC thermograms are shifted along the *y*-axis for comparison and labelled with their respective ion contents.

Most ion-containing materials of this study exhibit two endothermic transitions by means of DSC. Figure 7 below illustrates the DSC traces of C4ASA-films that are crosslinked with 2% BudMA. The DSC traces of the other C4ASA-films are given in Supplementary Figure 17. The observation of two transitions supports the DMA results. As discussed above with the DMA results, the low temperature transition was ascribed to the ion poor matrix and the high temperature transition to the ionic clusters. Similarly to the observations with DMA, the low temperature transition weakens and the high temperature transition becomes more prominent with increasing ion content. In fact, at the highest ion content, 30%, the low temperature transition can no longer be observed with DSC.

In the first heating run, a glass transition was observed approximately at −40°C, and an endothermic peak was observed at higher temperatures. In the second heating run, the low temperature endotherm remains virtually unchanged, while the high temperature endotherm weakens considerably. If a sample is kept at room temperature after the heating-cooling cycle, the high temperature endotherm starts to recover. DSC traces of a film with 2% BudMA and 30% C4ASA were measured 5 min and then 3 days after the first cooling, and finally 91 days after the previous heating run. Supplementary Figure 18 shows that the high temperature endotherm is weak when the sample is reheated 5 min after the first cooling. After keeping the sample at room temperature for 3 days, the endotherm restores partially. Finally, 91 days after the previous heating run the endotherm is even more prominent than for the as-prepared material. Integration of the endotherms gave a transition enthalpy of 28.6 J/g for the as-prepared material. Aging at room temperature for 5 min, 3 days, and 91 days yielded enthalpies of 1.5 J/g, 19.6 J/g, and 49.4 J/g, respectively. The increased ∆*H* is ascribed to enthalpy relaxation resulting from aging. Apart from aging, potential ways to manipulate the ionic crosslinking include *e.g.* solution ionic strength and pH.

Disappearance and gradual reappearance of the cluster transition has been observed before for ionomers (Tadano et al., 1989; Suchocka-GaŁaś, 1994; de Almeida and Kawano, 1999). de Almeida *et al.* attributed the disappearance and reappearance of the cluster transition to the time dependence of the relaxation process. The order of the ionic clusters is disrupted during the first heating run, which is seen as an endothermic transition. If the time between the first and second heating runs is short, the system has no time to regain its equilibrium state and endotherm is not observed in the second heating run. That said, if the first heating is followed by a long enough time for the system to reach conformational equilibrium, the high temperature endotherm is observed also in the second heating run (de Almeida and Kawano, 1999).

### The Thermal Phase Transitions’ Dependence on the Dynamic Crosslinker.

**FIGURE 8:**
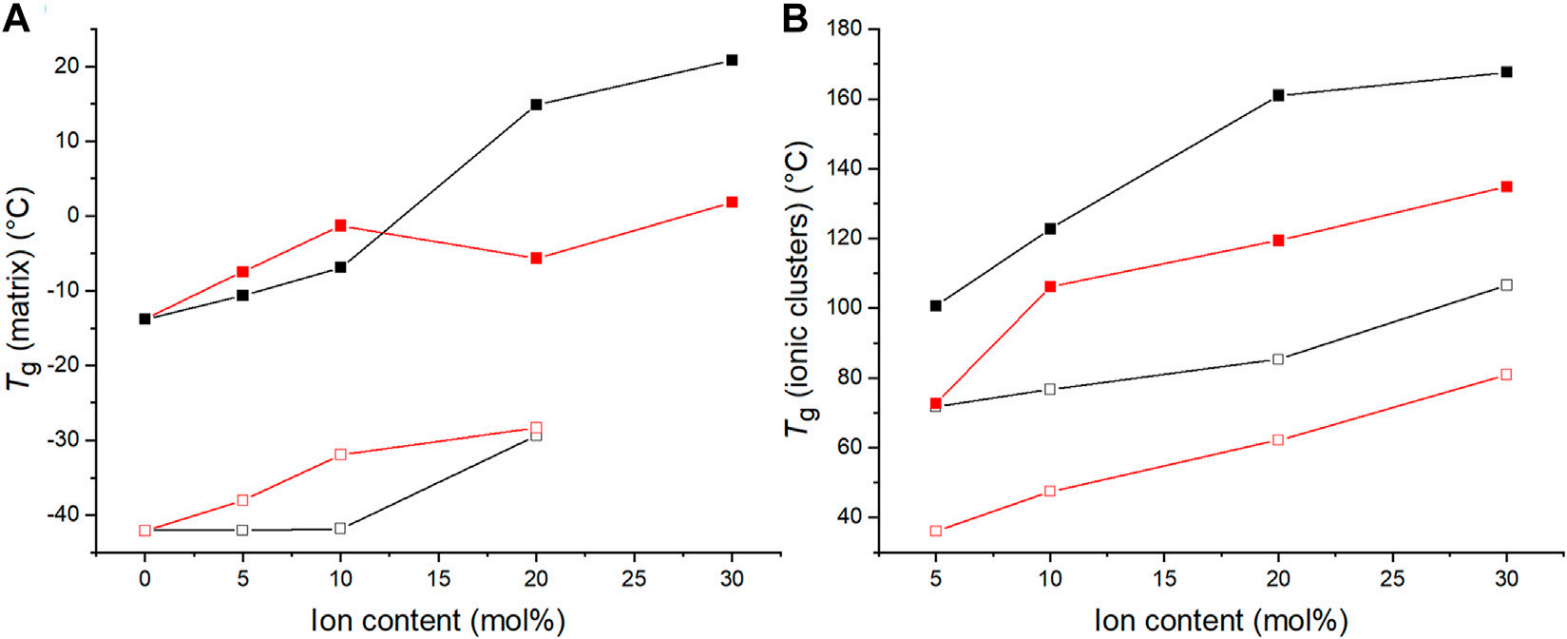
The glass transition temperatures of C4ASA- (black) and C6ASA-films (red) determined by means of DMA (filled symbols) and DSC (empty symbols). All the films contain 2% crosslinker. The glass transition temperatures are defined as the peaks of tan δ curves and the endotherms’ inflection points in DSC thermograms. **(A)** the *T*_g_ of the matrix and **(B)** the *T*_g_ of ionic regions.

Figure 8 compiles the glass transition temperatures of C4ASA- and C6ASA-films. Both materials’ *T*_g_s increase with increasing ion content. As was discussed above for the C4ASA-films, the dynamic crosslinks in the ionic clusters restrict chain movements, thus delaying the glass transitions (Eisenberg et al., 1990). Compared to C4ASA-films, C6ASA-films have generally lower glass transition temperatures. C4ASA and C6ASA differ only in their alkyl chains in the amine group, with C4ASA having butyl chains and C6ASA hexyl chains. The longer side chains of C6ASA result in clusters with lower ion densities. Hence, C6ASA yields lower glass transition temperatures than C4ASA. Furthermore, since C4ASA has shorter alkyl chains, it has fewer possible configurations and less mobility than C6ASA (Eisenberg et al., 1990). The alkyl chains can also lower the frictional interaction between neighbouring chains and act as plasticizers (Visser and Cooper, 1991). Generally factors which increase the volume disturbed by a rotating segment or increase the density of the solid increases *T*_g_ (Bueche, 1953). Although C6ASA is larger, it is less stiff than C4ASA and exhibits thus lower glass transition temperatures. However, the size difference between C4ASA and C6ASA is small and hence the strong plasticization effect of C6ASA is mainly ascribed to its lower charge density.

At high ion content, in relation to the matrix peak, the cluster peak was more prominent with C4ASA than with C6ASA. Kim et al. (1999b) proposed that groups that have different contact surfaces form differently sized multiplets (Kim et al., 1994b). The contact surface is the area of the segments that are in contact with the multiplet (Kim et al., 1994b). This includes the ionic moieties as well as portions of the matrix polymer chains that are included in the multiplet. The radius of the contact surface area is inversely related to the radius of the multiplet (Kim et al., 1994b). Therefore, for the same ion content, C4ASA forms fewer, but larger multiplets than C6ASA. The larger clusters can interact more strongly with the surrounding matrix. Thus, C4ASA gives a more prominent cluster *T*_g_ peak than C6ASA. It is worth noting that the polymerization of the ionic moieties occurred concurrently with the chemical crosslinks of the butyl acrylate matrix. Due to the confined environment, predictions of the clusters’ size and composition, cannot be made using conventional models. Nonetheless, the differences in mechanical behavior, which will be discussed later, together with the DMA results support the conclusion that C4ASA and C6ASA result in different cluster microstructures.

**FIGURE 9:**
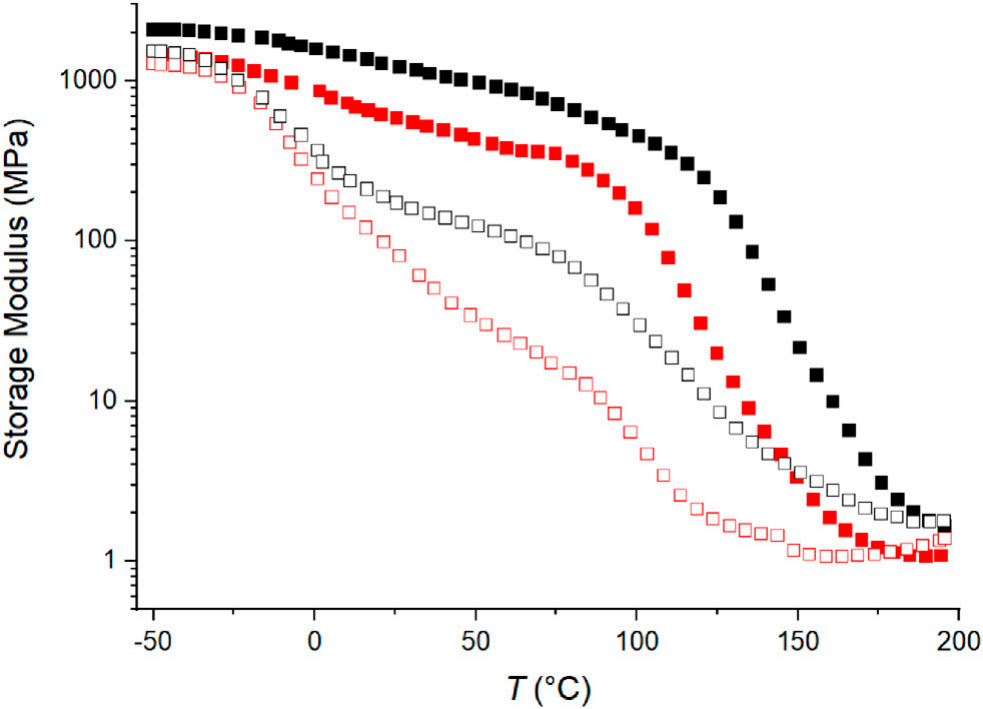
The storage moduli as a function of temperature of films with 30% (filled symbol), and 10% (empty symbol) ion content. C4ASA-films are depicted with black symbols and C6ASA-films with red symbols. Each film is chemically crosslinked with 2% BudMA.

As discussed above for the C4ASA-films, the storage modulus is related to crosslink density. Figure 9 compares the storage moduli of films that contain different ionic comonomers – C4ASA and C6ASA. For clarity, only two ion contents (10 and 30%) are depicted. Crosslink density is higher for films with C4ASA. This supports the interpretation that compared to C4ASA-clusters, the longer hydrocarbon side chains in C6ASA result in clusters with lower ion density. The storage moduli also show that the films prepared with C6ASA start to flow at lower temperatures than the films that were prepared with C4ASA.

Overall, the thermal behaviour of C4ASA- and C6ASA-containing materials is similar, but their phase transitions occur at slightly different temperatures. The plasticizing effect of the longer alkyl side chains shifts the glass transition temperature of C6ASA-containing materials to lower temperatures compared to analogous C4ASA-containing materials.

**FIGURE 10:**
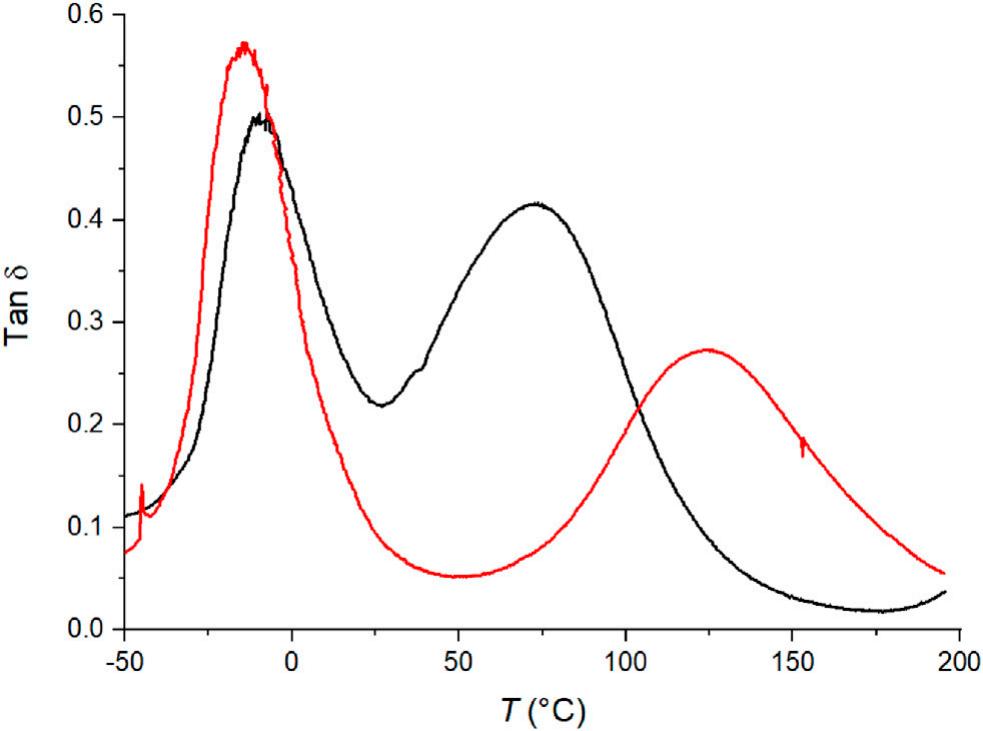
The tan δ *versus* temperature of films with 5% C6ASA (black) and 5% PC6ASA (red). Both films are chemically crosslinked with 2% BudMA.

One film was prepared using polymerized C6ASA (PC6ASA) as the ionic moiety. The film was made with the amount of polymer corresponding to 5 mol% of repeating units in the feed. Higher polymer-contents could not be used as PC6ASA was not soluble enough in butyl acrylate. Figure 10 illustrates the tan δ curves of films prepared with 5% C6ASA and 5% PC6ASA. The film that was prepared with the polymer has lower peak2/peak1 ratio than the one prepared with the monomer. The peak separation is also more pronounced with PC6ASA. The fact that the intensity of the matrix peak is slightly higher for the PC6ASA-film than for the C6ASA-film indicates that the PC6ASA-film has a lower volume fraction of material of restricted mobility than a similar film that has been prepared with C6ASA. Since the PC6ASA-film was prepared by adding polymer to the feed, the material is more block-like than the C6ASA-film, *i.e.* the clusters and the matrix are more separated. The higher degree of separation between the filler and matrix means that the clusters of the polymer-filled film are more densely packed as they contain less matrix polymer groups. Therefore, the PC6ASA-film contains a smaller fraction of regions of restricted mobility. It is worth noting that the polycation has polymerizable counterions. As the counterions polymerize under UV illumination, they set the polyelectrolyte in the butyl acrylate matrix through attractive Coulombic interactions.

The use of PC6ASA and C6ASA also resulted in different glass transition temperatures. The high temperature, cluster related *T*_g_ was higher when PC6ASA was used in the feed. The *T*_g_ of the polymer-filled film was close to the *T*_g_ of a film that was prepared with 20% C4ASA. This supports the rationalization that the clusters of the C6ASA-film (as opposed to the PC6ASA-film) contain more matrix polymer groups that lower the cluster *T*_g_ and thus they are copolymer-like rather than a mixture of two homopolymers. C4ASA homopolymer was not soluble in butyl acrylate and thus was not used in film preparation.

### Tensile Properties of C4ASA Films

The previous sections discussed the thermal properties of the materials. The mechanical properties’ dependence on chemical and dynamic crosslinking was studied by means of stress-strain measurements.

**FIGURE 11:**
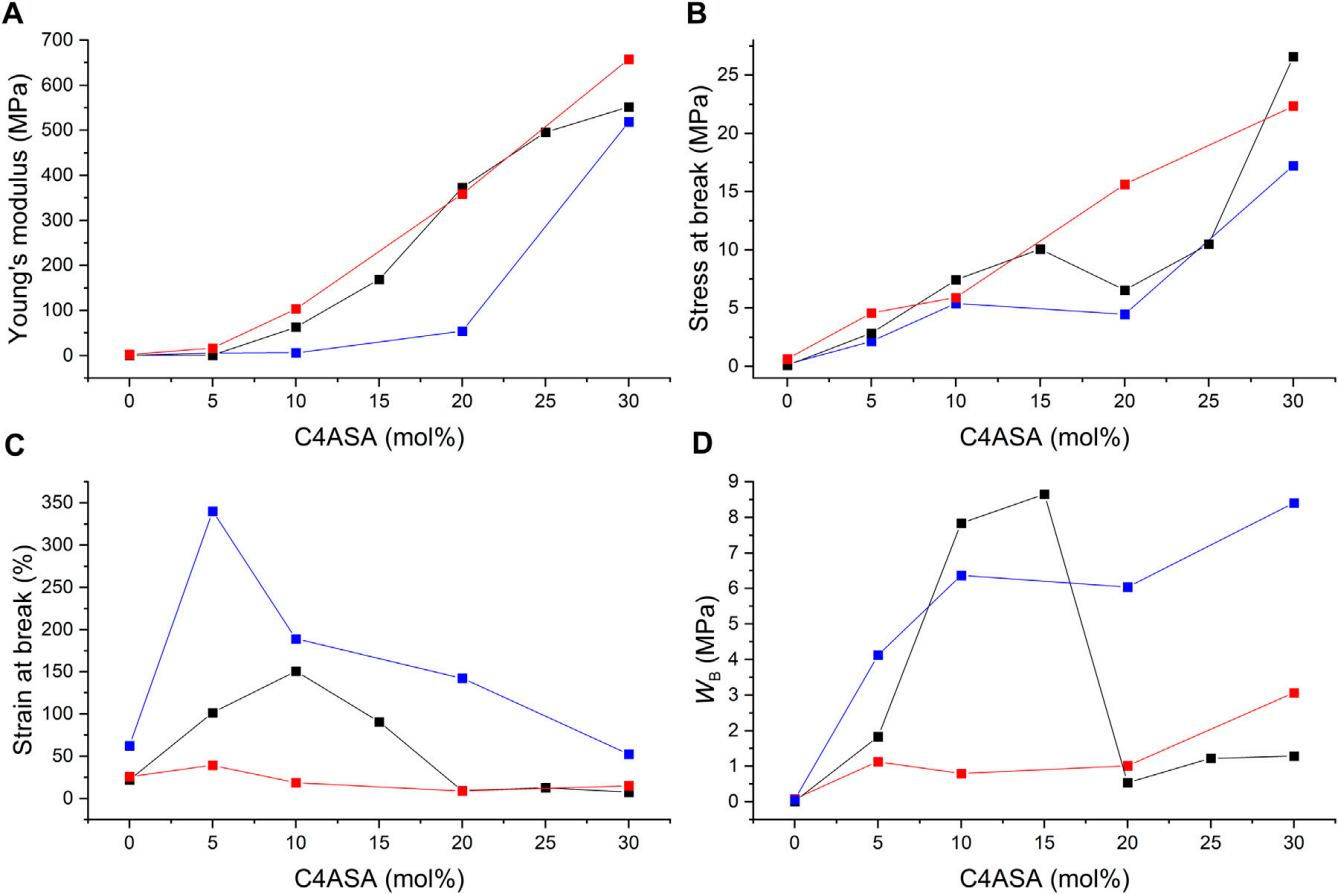
The ion content dependence of **(A)** Young’s modulus, **(B)** stress at break, **(C)** strain at break, and **(D)** fracture energy (WB) of C4ASA-films with 1% (blue), 2% (black), and 5% (red) BudMA. The fracture energies were defined as the integrals of stress-strain curves.

Stress-strain curves were recorded for all films, and their Young’s moduli, stresses at break, and strains at break were determined and plotted as a function of the ion content. In addition, the fracture energies (*W*_B_), *i.e.* the energy per unit volume needed to break the samples were calculated by integrating the stress-strain curves. Fracture energy is a measure of toughness (Zosel and Ley, 1993). Figure 11 illustrates the results of the films with C4ASA as the dynamic crosslinker. The stress-strain curves have been given in Supplementary Figure 19.

Stress at break and the Young’s modulus rise steadily with increasing concentration of ionic crosslinker (Figure 11A,B). Copolymerization with ions strengthens the materials considerably. For instance, addition of 30% C4ASA yields a 970-fold increase in stiffness and 250-fold increase in strength compared to a non-ionic material. The electrostatic interactions of the oppositely charged groups of C4ASA offer reversible crosslinks that can break and reform under stress (Luo et al., 2015). These reversible crosslinks toughen the material by acting as sacrificial bonds that dissipate energy as they break (Neal et al., 2015).

Chemical crosslinking affects the elastic modulus, as seen in Figure 11A. The materials with the lowest concentration of BudMA are the least stiff and the rigidity increases with the crosslink density. The material strength is also affected by chemical crosslinking. The effect is subtle, and the differences are the most evident at higher ion contents. The material strength appears to increase with increasing BudMA-content. The materials with 1% BudMA are the weakest throughout the studied ion content range. This is an indication of the fact that crosslinks between polymer chains hinder the development of cracks (Fischer et al., 1995). The most highly crosslinked materials are the strongest at all ion contents apart from 30%.

The strain at break of the films increases with small amounts of dynamic crosslinker but starts to decrease when the concentration of C4ASA is increased further (Figure 11C). The 2% BudMA films are the most elastic with 10% of dynamic crosslinker, whereas the most elastic 5% BudMA films are obtained with 5% of C4ASA. The strain at break of the films with 1% of BudMA decreases steadily with increasing ion content after reaching peak elasticity at 5% ion content. The decrease of the strain at break can be ascribed to the formation of ionic clusters at high ion contents. Since the dynamic crosslinker forms ionic clusters that act as physical crosslinks, they reduce the chain mobility and, consequently, the strain at break (Mohanty et al., 1997).

Evidently, the loss of chain mobility arising from chemical crosslinks results in decreased elasticity. The films with 1% crosslinker are the most elastic, whereas the materials that contain 5% crosslinker exhibit the lowest strains at break.

Toughness of a material relates to its resistance against crack growth – it indicates how much energy a material can absorb before rupturing (Ligon-Auer et al., 2016). Hence, for the material to be tough, it needs to withstand high stress and strain. This means that the material needs the right balance between strength and extensibility. The fracture energies of the films suggest that increasing the number of chemical crosslinks decreases the toughness of the material (Figure 11D). The films with 1 and 5% crosslinker are the toughest with 30% C4ASA, which was the highest ion content studied. The toughness of the films with 2% crosslinker increases first, reaching its peak with 15% of C4ASA, and decreases as the concentration of ionic monomer is further increased. The 2% BudMA-films’ decreased ductility at high ion contents is seen as a decrease in toughness.

### The Tensile Properties of Materials With Different Dynamic Crosslinkers

**FIGURE 12:**
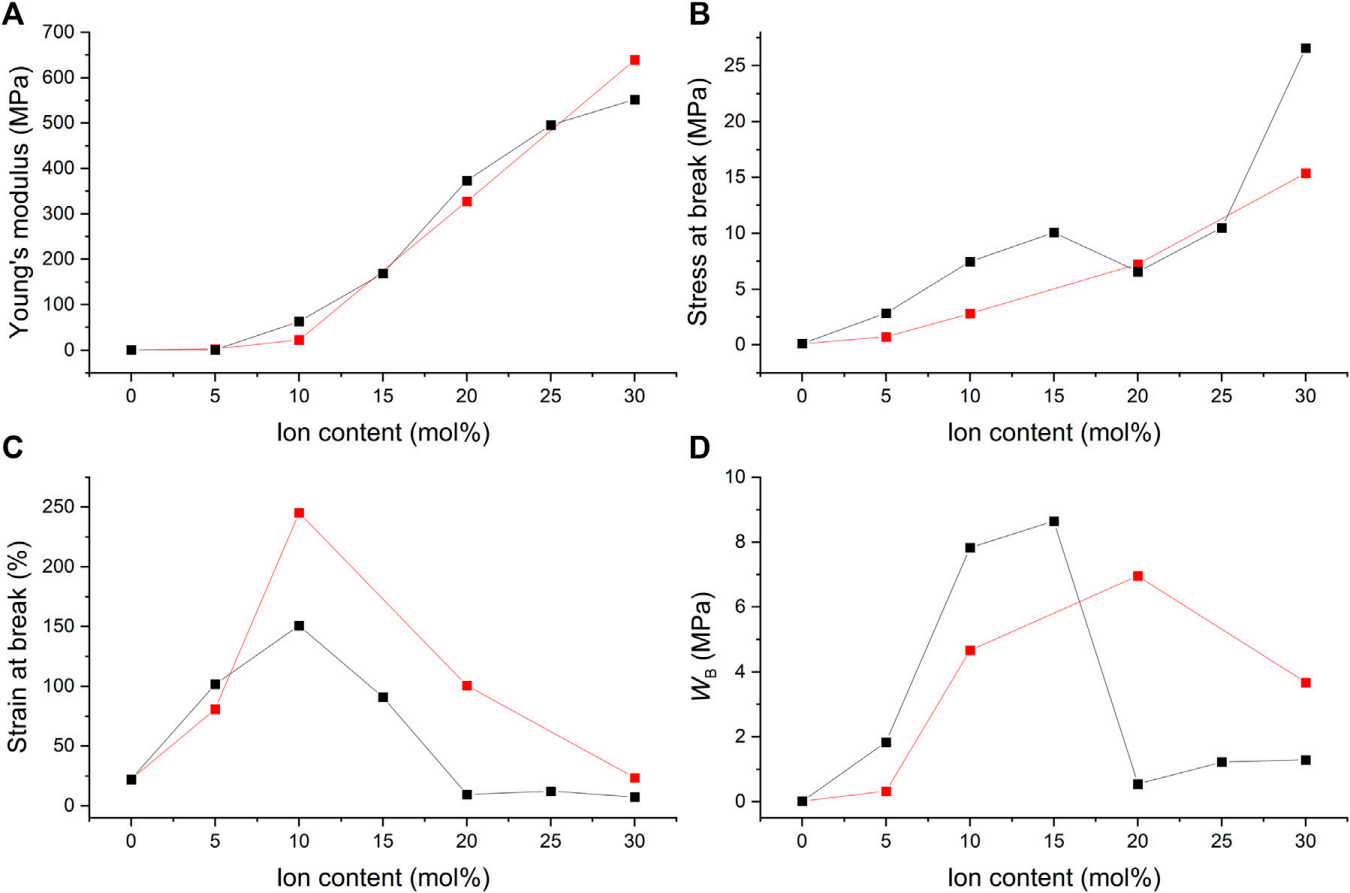
The ion content dependence of **(A)** the Young’s modulus, **(B)** the stress at break, **(C)** the strain at break, and **(D)** the fracture energy (WB) of films with 2% BudMA and varying concentrations of C4ASA (black) or C6ASA (red). The fracture energies were defined as the integrals of stress-strain curves.

Figure 12 compares the mechanical properties of the films that contain varying concentrations of different ionic crosslinkers, C4ASA or C6ASA. C4ASA yields slightly stiffer and stronger materials than C6ASA does possibly due to its higher ion density (Figure 12A,B). C6ASA differs from C4ASA only in the amine side groups. The longer hydrocarbon side chains in C6ASA give clusters with lower ion density than C4ASA. Therefore, it is likely that C6ASA-containing materials have a lower density of ionic groups that impart strength and stiffness to the material (Visser and Cooper, 1991). In addition, since C6ASA has longer hydrocarbon chains, it is reasonable to assume that the repeating unit is more compatible with the nonpolar matrix than C4ASA is. Improved compatibility with the matrix can result in smaller ion aggregates that form weaker crosslinks (Visser and Cooper, 1991).

The Young’s modulus of the C4ASA- and C6ASA-films as a function of ion content show a change in slope when the ion content exceeds 5% (Figure 12A). The change in the slope is ascribed to changes in the microstructure of the ionic aggregates. At low ion contents, smaller ionic aggregates, multiplets, dominate and act as weak ionic crosslinks (Bellinger et al., 1994). When a critical ion content is surpassed, clusters dominate. Not only do the clusters serve as crosslinks that are more efficient, they also form a second phase that acts like a reinforcing filler (Bellinger et al., 1994). However, the stress at break as a function of ion content does not exhibit such change in slope.

C6ASA-containing films are more elastic than C4ASA-containing films (see Figure 12C). As discussed above, the C4ASA-films have higher ion density compared to the C6ASA-films. The dynamic crosslinker forms physical crosslinks that reduce chain mobility and lower the strain at break. Since the C6ASA-materials have lower ion densities and higher mobilities, they have higher strains at break compared to the C4ASA-materials. The strains at break increase up until to 10%. Both C4ASA and C6ASA give the highest elongations at 10% ion content. Further increases in the ion content are detrimental to the strain at break due to high density of dynamic crosslinks.

Figure 12D compares the fracture energies of the films with 2% crosslinker, and varying amounts of C4ASA or C6ASA. The C4ASA-films are the toughest with 15% dynamic crosslinker, whereas the toughest C6ASA-films are obtained with 20% of the ionic monomer in the feed. At the low ion contents, C4ASA yields tougher films than C6ASA. In contrast, at the high ion contents C6ASA-films turn out to be tougher. This suggests that even though C4ASA-films are slightly stronger, the high elasticity of C6ASA-films offsets the lower strength. Therefore, the C6ASA-films are tough at high ion contents as the number of charges per unit volume is lower than in C4ASA due to the higher molecular weight of the former.

In summary, both dynamic crosslinkers provide significant improvements to the mechanical properties of chemically crosslinked butyl acrylate. The improvements in strength and stiffness are similar with both C4ASA and C6ASA. However, C6ASA yields more elastic materials.

Supplementary Figure 20 illustrates the mechanical properties of three films that contain similar concentrations of different ionic crosslinkers: C4ASA, C6ASA, or PC6ASA. The film that contains 5% PC6ASA, *i.e.* polymerized C6ASA, is more tough and strong than a corresponding film with C6ASA. However, compared to the film with C4ASA, the polymer-filled film is weaker, less ductile, and less tough. The polymer-filled film is more block-like than the film that was prepared with C6ASA, as was discussed above for the DMA results. Since the formation of hard and rigid ionic clusters or aggregates is the reason for the increased strength of ion-containing materials, it is reasonable to deduce that the more ordered clusters of the polymer-filled film result in more effective strengthening the matrix. The clusters of the C6ASA-film are weaker since they contain more matrix polymer chains than the clusters of the polymer-filled material.

## Conclusion

Butyl acrylate-based materials were toughened successfully through ionic interactions. Dynamic crosslinkers, salts with polymerizable cations and anions, were used to physically crosslink butyl acrylate. The ion pairs of dynamic crosslinkers act as sacrificial bonds that toughen the material. A variety of materials was prepared and characterized via dynamic mechanical analysis and thermogravimetric analysis. The toughening effect of chemical and dynamic crosslinking was evidenced through tensile testing.

Combining chemical and dynamic crosslinks resulted in tough materials that required much energy to be broken. 4- (Tributylammoniomethyl)styrene sulfopropyl acrylate (C4ASA), and 4-(trihexylammoniomethyl)styrene sulfopropyl acrylate (C6ASA) were used as the dynamic crosslinkers. Chemical crosslinker was used to bring shape stability.

The incorporation of dynamic crosslinker results in appreciable enhancements in strength, stiffness, elasticity, and toughness. The enhanced mechanical properties can be rationalized to arise from ionic clusters in which the dynamic crosslinkers form reversible physical crosslinks. The ionic interactions have a significant effect on the strength and stiffness of the materials. The Young’s modulus and the stress at break increase steadily with added ions. Even the strain at break increases at first but starts to decrease when the ion content is increased further. For example, the C4ASA-films exhibit peak elasticity with 5–10 mol% ion content.

Dynamic mechanical analysis and differential scanning calorimetry show that the materials contain phase-separated ionic regions and exhibit at least two relaxations. The first one arises from the glass transition of the matrix and the later ones from ionic clusters. Both measurement techniques revealed that both matrix and cluster *T*_g_s increase with increasing ion content. The shifts in *T*_g_ reflect the ionic domains’ ability to act as crosslinks that restrict chain movement.

The dynamic crosslinkers’ (C4ASA and C6ASA) effect on the thermal phase transitions and the tensile properties were compared. The Young’s moduli and stresses at break were slightly lower with C6ASA than they were with C4ASA. However, C6ASA-films were more elastic. Dynamic mechanical analysis showed that C4ASA forms larger but fewer multiplets than C6ASA did. Larger aggregates can form more effective crosslinks. Both C4ASA and C6ASA exhibited increased *T*_g_s with increasing ion content. However, C6ASA had lower *T*_g_s than C4ASA. The differences can be ascribed to the cluster microstructure. Since C6ASA contains hexyl chains as opposed to the butyl chains of C4ASA, copolymerizing with C6ASA results in materials with lower ion density and higher chain mobility.

The material properties of films that were prepared with a monomer (C6ASA), or a corresponding polymer (PC6ASA) were investigated. Using a polymer resulted in a more block-like structure with a high cluster *T*_g_. The monomer film had a lower cluster *T*_g_ since its clusters contained larger portions of matrix polymer groups. Not only did the monomer-film have a lower *T*_g_, it was also weaker and less tough than the polymer-filled film. The cluster structure was inferred to be the reason behind the observed differences. The hard and rigid clusters of the polymer-filled film strengthened the matrix more effectively. The comparison between the monomeric and polymeric forms reveal that in the films made from monomers only, the phases do not separate completely. Rather phases that contain practically only BuA and other phases that contain BuA along with dynamic crosslinker form.

This study introduces a novel method to improve the mechanical properties of butyl acrylate based photopolymerization resins using salts that have a polymerizable double bond both in the cation and in the anion as ionic crosslinkers. The ionic crosslinker clearly altered the mechanical properties in a beneficial manner and the approach is generalizable to other monomers as well. The methodology will open new avenues for development of mechanically durable materials for photopolymerization applications.

## Data Availability

The original contributions presented in the study are included in the article/Supplementary Material, further inquiries can be directed to the corresponding authors.
