# Corrigendum: Tough materials through ionic interactions

**DOI:** 10.3389/fchem.2023.1241556

**Published:** 2023-07-03

**Authors:** Linda Salminen, Erno Karjalainen, Vladimir Aseyev, Heikki Tenhu

**Affiliations:** ^1^ Department of Chemistry, University of Helsinki, Helsinki, Finland; ^2^ VTT Technical Research Centre of Finland Ltd., Espoo, Finland

**Keywords:** photopolymerization, dynamic crosslinker, crosslinking, reinforcement, tensile strength

In the published article, there was an error in Figures 11, 12. The order of magnitude was incorrect in Figures 11A, D and Figures 12A, D. The correct figures and their captions appear below.

In the published article, there was an error in **Supplementary Figure S20**, which had incorrect dimensions. The correct figure and its caption appear below.

**FIGURE 11:**
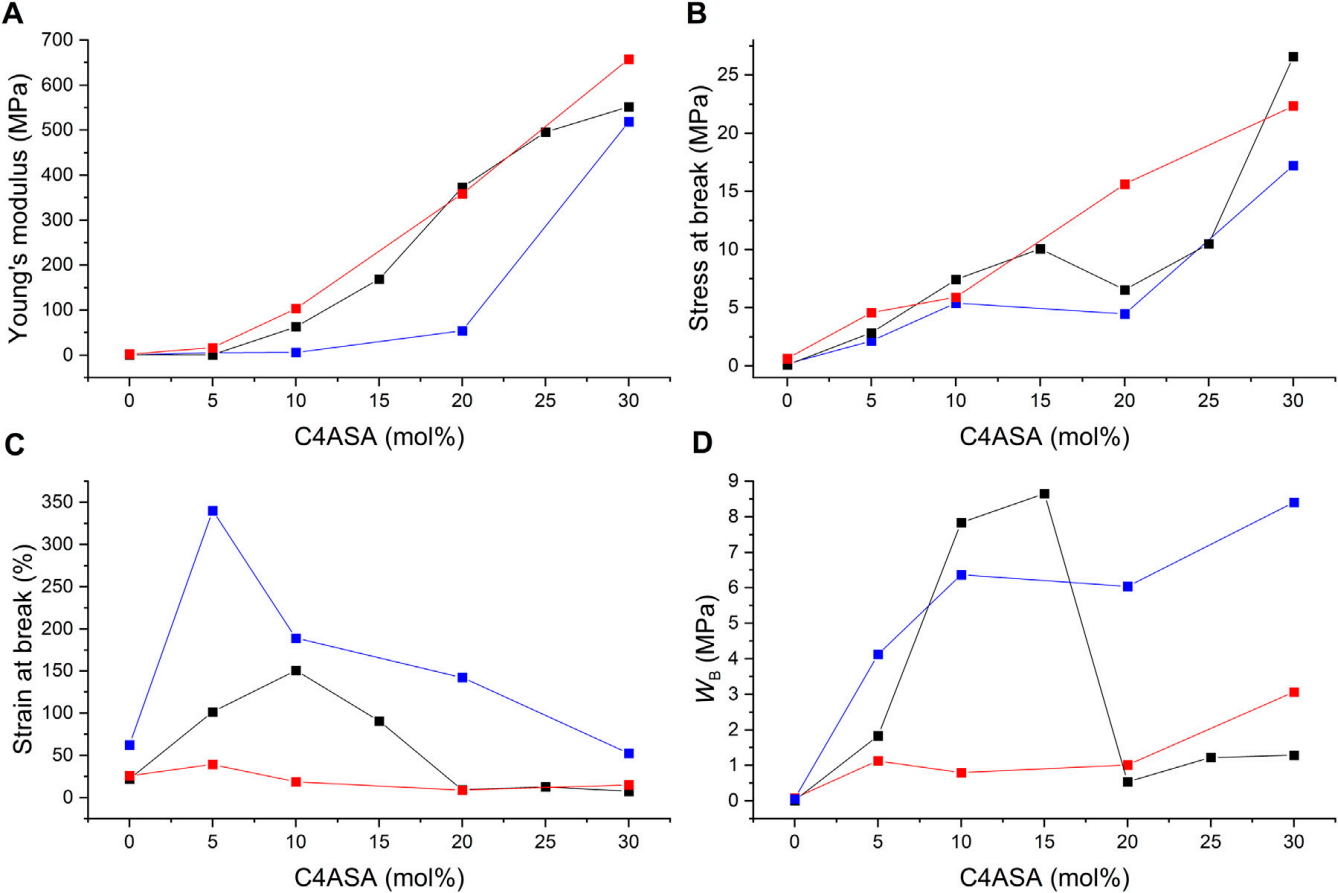
The ion content dependence of **(A)** Young’s modulus, **(B)** stress at break, **(C)** strain at break, and **(D)** fracture energy (WB) of C4ASA-films with 1% (blue), 2% (black), and 5% (red) BudMA. The fracture energies were defined as the integrals of stress-strain curves.

**FIGURE 12:**
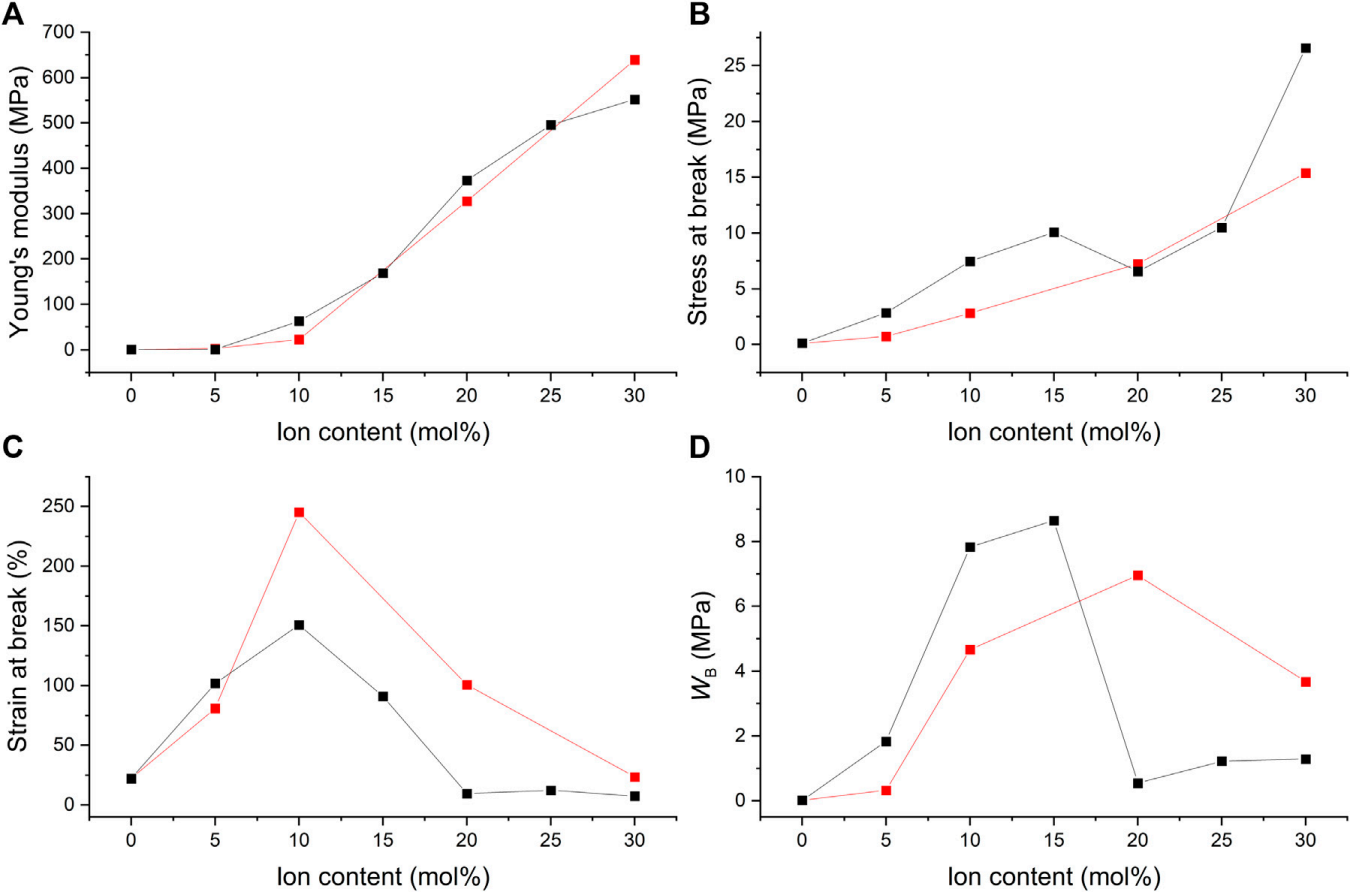
The ion content dependence of **(A)** the Young’s modulus, **(B)** the stress at break, **(C)** the strain at break, and **(D)** the fracture energy (W_B_) of films with 2% BudMA and varying concentrations of C4ASA (black) or C6ASA (red). The fracture energies were defined as the integrals of stress-strain curves.

**SUPPLEMENTARY FIGURE S20:**
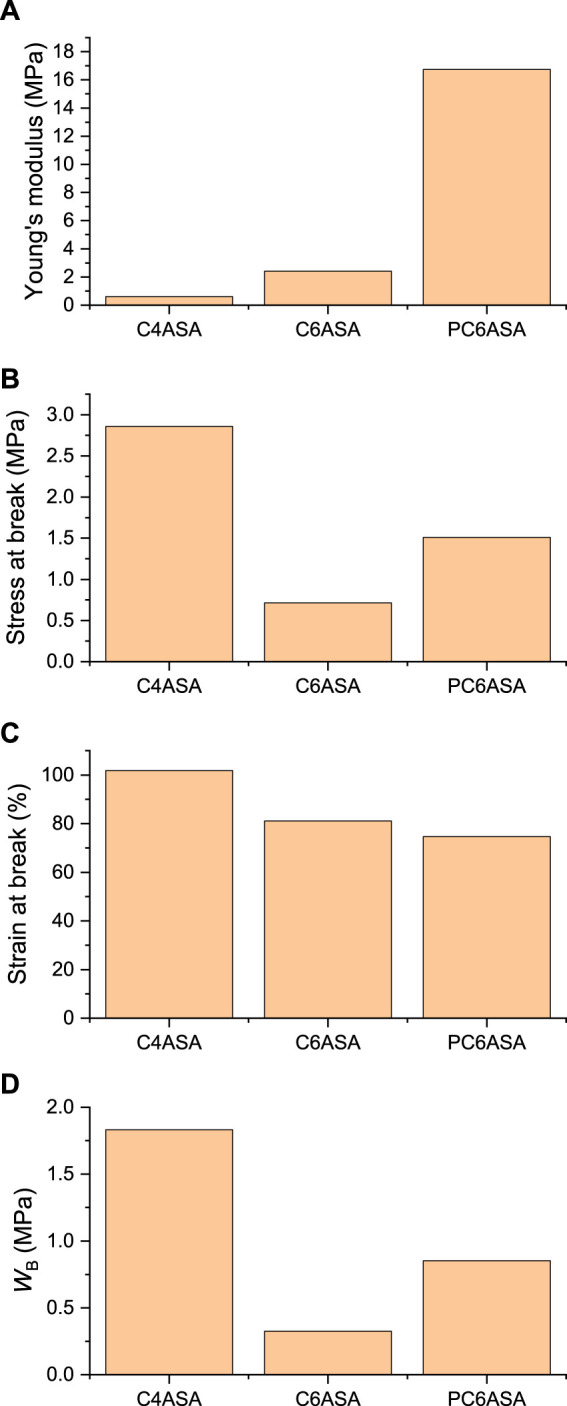
The Young’s moduli **(A)**, stresses at break **(B)**, strains at break **(C)**, and fracture energies **(D)** of films with 5% of C4ASA, C6ASA, or PC6ASA. Each film contains 2% BudMA.

The authors apologize for these errors and state that this does not change the scientific conclusions of the article in any way. The original article has been updated.

