# Supplementary material for: Tough Materials Through Ionic Interactions
Source: Front Chem. 2021 Jul 27;9:721656. doi: 10.3389/fchem.2021.721656 (PMC8354582; doi:10.3389/fchem.2021.721656)
Supplement: Supplementary file 1 [file DataSheet1.pdf]

Supplementary Material

1 NMR spectra of the dynamic crosslinkers

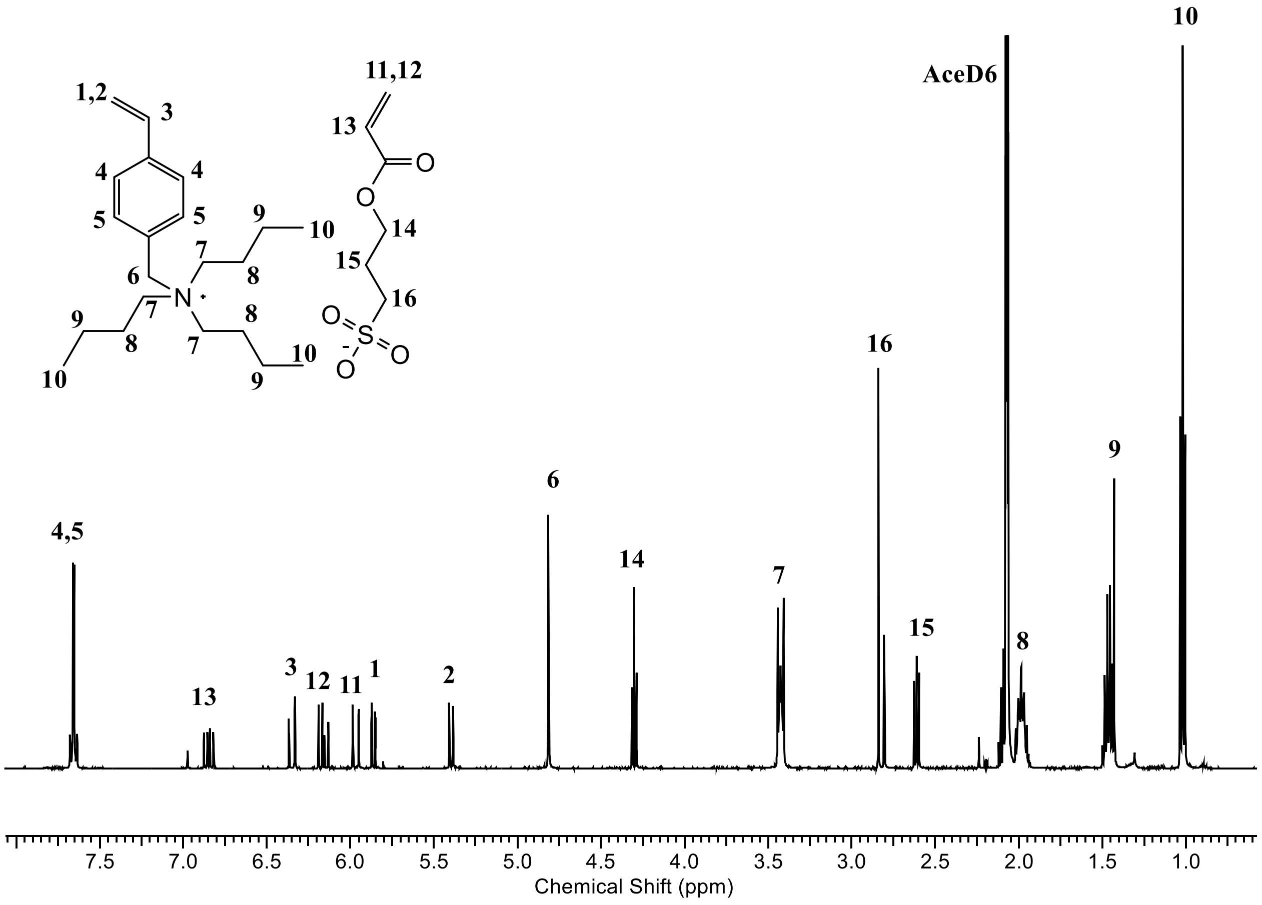

Supplementary Figure 1. The NMR spectrum of tributyl-(4-vinylbenzyl)ammonium sulfopropyl acrylate (C4ASA) in deuterated acetone (AceD6).

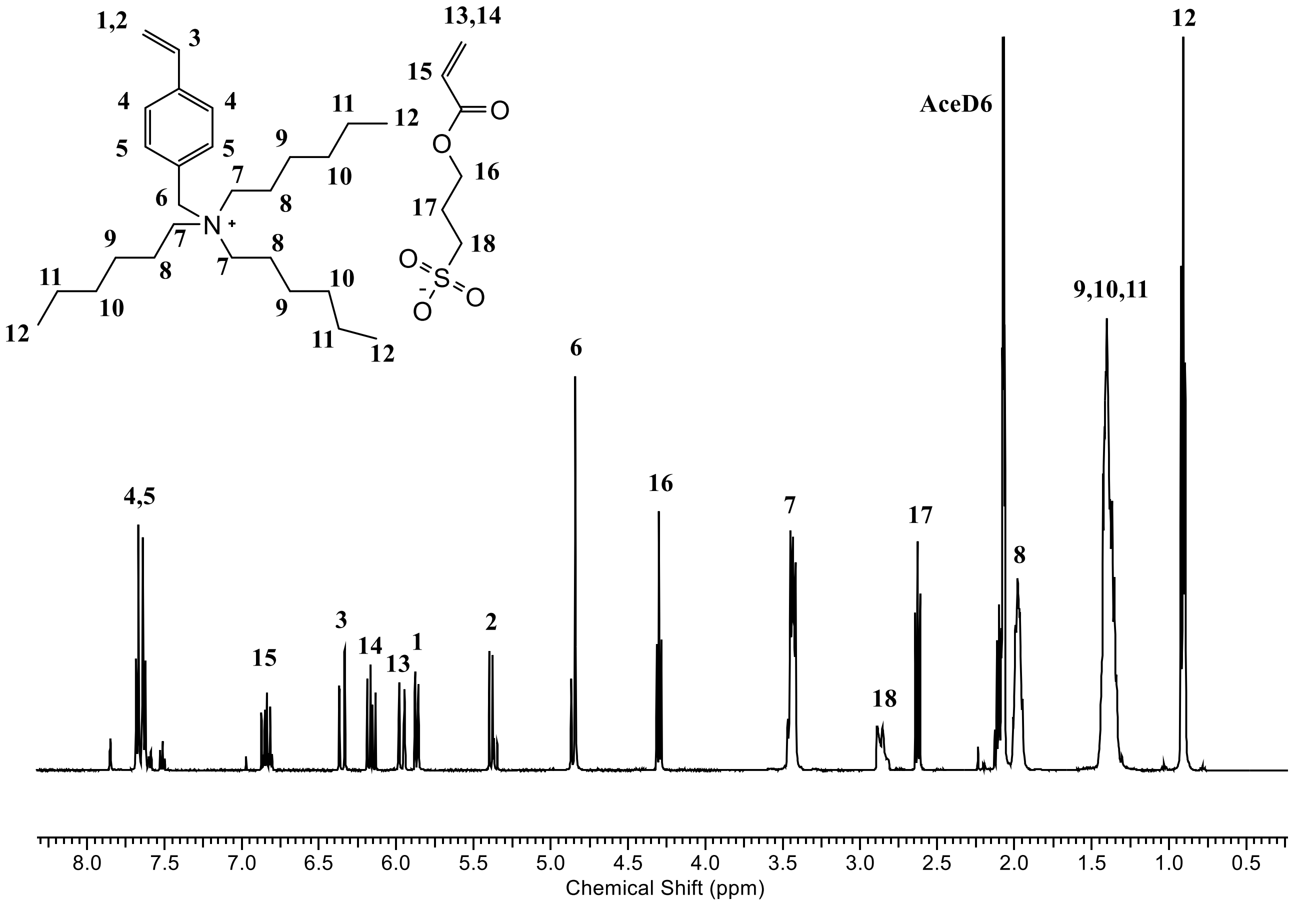

Supplementary Figure 2. The NMR spectrum of trihexyl-(4-vinylbenzyl)ammonium sulfopropyl acrylate (C6ASA) in deuterated acetone (AceD6).

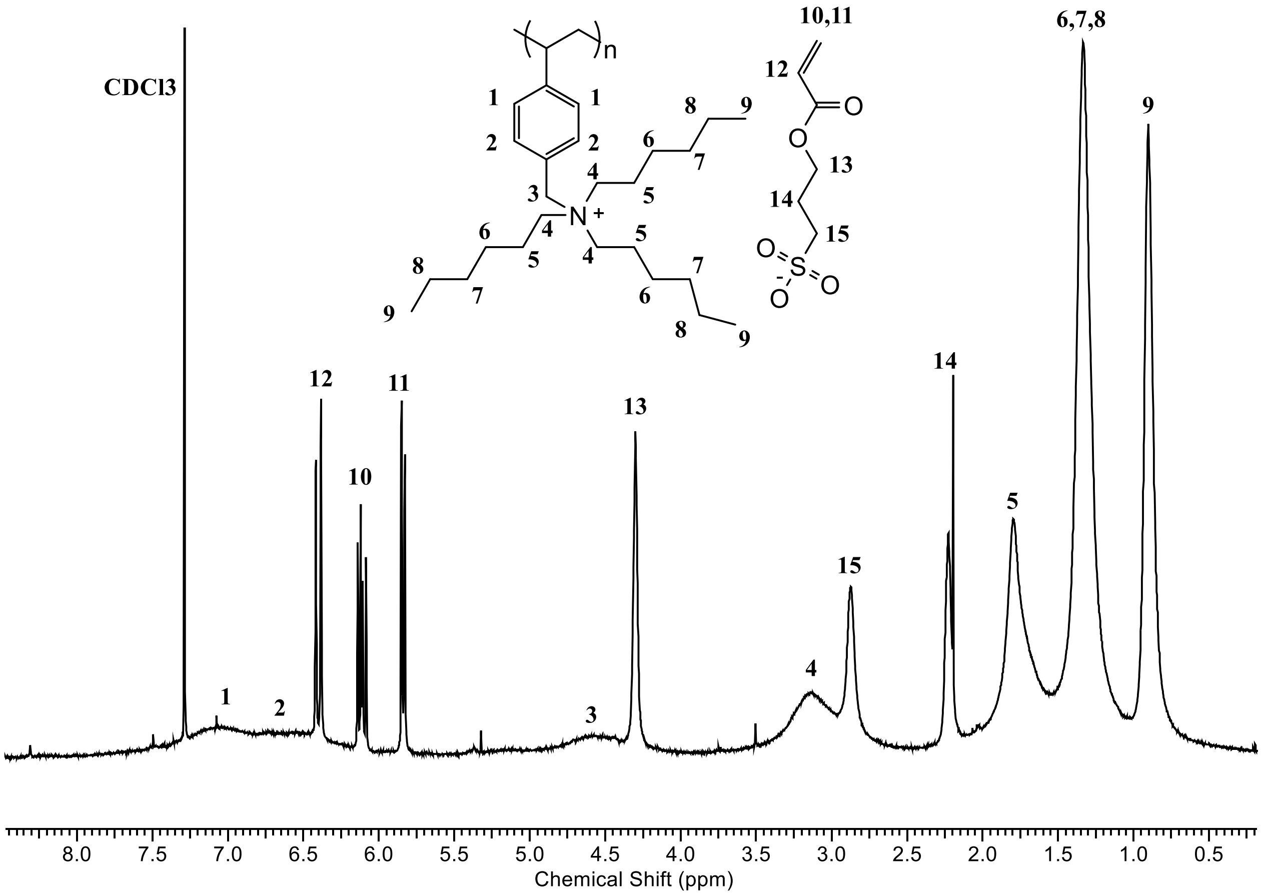

Supplementary Figure 3. The NMR spectrum of poly[trihexyl-(4-vinylbenzyl)ammonium sulfopropyl acrylate] (PC6ASA) in deuterated chloroform (CDCl3).

2 Thermal stability of the materials

The decomposition temperatures of the films were determined through thermogravimetric analysis and the obtained curves are presented in Supplementary Figures 4-7. The decomposition temperature gives information about the thermal stability of the materials. This information was used to determine a suitable temperature range for DMA measurements. The figures show that with increasing content of ionic monomer, the TGA curve becomes increasingly close to the curve of the corresponding homopolymer, *i.e.* the C4ASA-films' curves near the curve of polymerised C4ASA and C6ASA-films' curves near the TGA curve of PC6ASA.

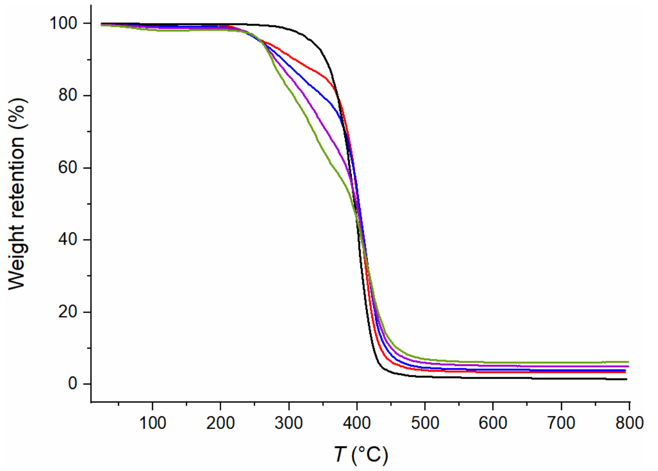

Supplementary Figure 4. TGA-curves of films with 5% BudMA and varying amounts of C4ASA. The C4ASA-contents of the films are 0% (black), 5% (red), 10% (blue), 20% (purple), and 30% (green).

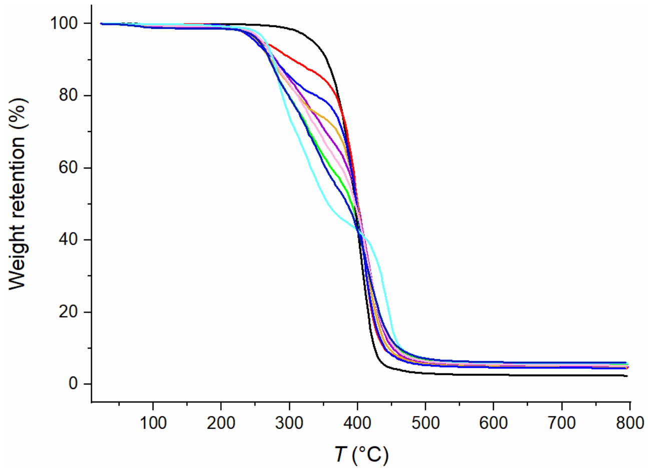

Supplementary Figure 5. TGA-curves of films with 2% BudMA and varying amounts of C4ASA. The C4ASA-contents of the films are 0% (black), 5% (red), 10% (blue), 15% (orange), 20% (violet), 25% (pink), and 30% (green). The pale blue curve corresponds to pure polyelectrolyte, PC4ASA.

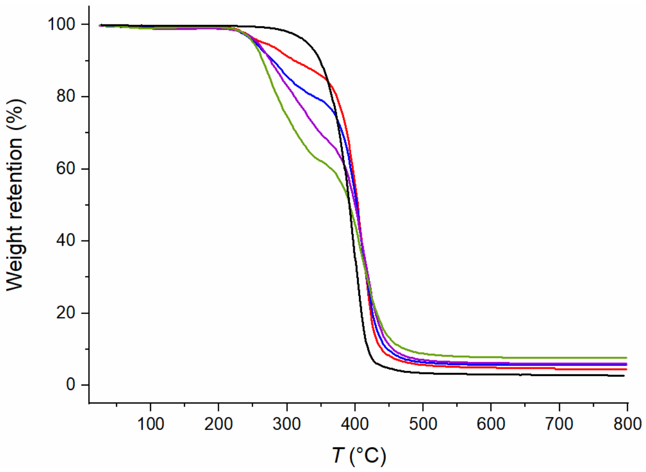

Supplementary Figure 6. TGA-curves of films with 1% BudMA and varying amounts of C4ASA. The C4ASA-contents of the films are 0% (black), 5% (red), 10% (blue), 20% (purple), and 30% (green).

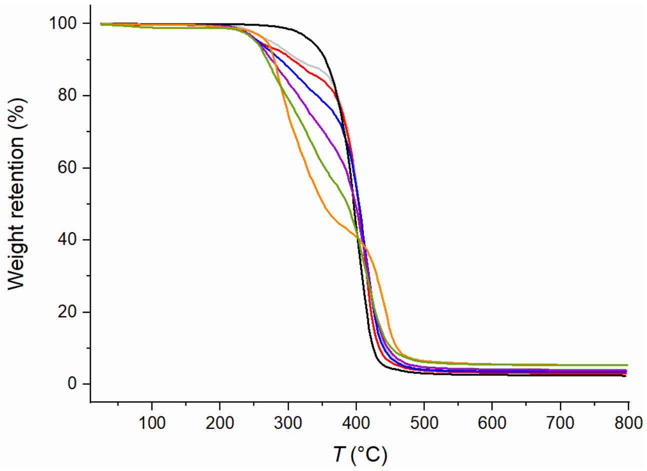

Supplementary Figure 7. TGA-curves of films with 2% BudMA and varying amounts of C6ASA. The C6ASA-contents of the films are 0% (black), 5% (red), 10% (blue), 20% (purple), and 30% (green). Additionally, the TGA-curves of PC6ASA (orange) and a butyl acrylate film with 2% BudMA and 5% PC6ASA (grey) have been depicted.

Supplementary Figure 8 is a depiction of the degradation onset temperatures of C4ASA-films. The films with 5% crosslinker are the most stable. High crosslink density hinders the diffusion of volatiles, hence delaying the degradation. With low concentrations of ionic monomer, the films with 2% crosslinker are thermally the least stable. However, it is worth noting that the thermal stability of the ionomers is lower than the thermal stability of butyl acrylate without an ionic comonomer. For example, butyl acrylate that has been crosslinked with 2% BudMA starts to degrade at 310 °C.

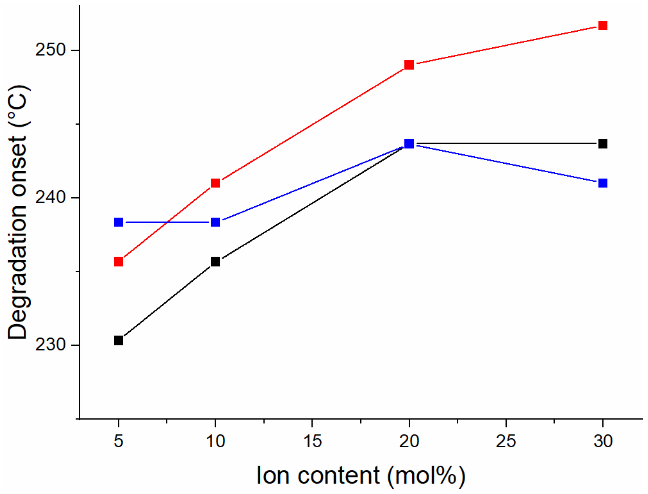

Supplementary Figure 8. The degradation onsets of the C4ASA-films with 1% (blue), 2% (black), and 5% (red) of chemical crosslinker BudMA as a function of ion content. The degradation onset was defined as the temperature where – starting from 150 °C – 2% of the material had degraded. The percentages (ion content and crosslinker content) are the concentrations of the respective monomers in the feed.

Comparison of the TGA curves of C6ASA-, C4ASA-, and PC6ASA-films reveals subtle differences in the onsets of degradation (Supplementary Figure 6). As the ionic content of the material increases, the degradation temperature increases ever so slightly, nearing the degradation temperature of corresponding pure polyelectrolyte. The film that contained 5% of PC6ASA was the most stable of the ionic films as its degradation temperature was close to that of pure PC6ASA. C4ASA-films are slightly more thermally stable than the films with C6ASA.

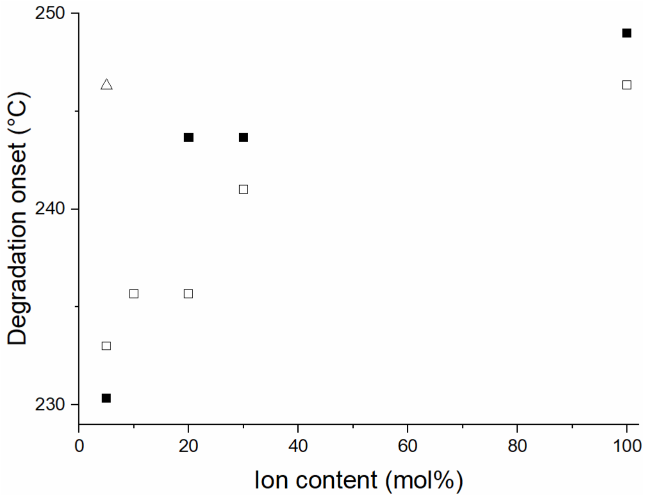

Supplementary Figure 9. The degradation onset temperatures of films with 2% chemical crosslinker and varying amounts of C4ASA (■), C6ASA (□), and PC6ASA (△). The points at 100% ionic monomer concentration are an exception as they belong to pure polyelectrolytes, not crosslinked films. The degradation onset was defined as the temperature where – starting from 150 °C – 2% of the material had degraded.

3 Thermal phase transitions

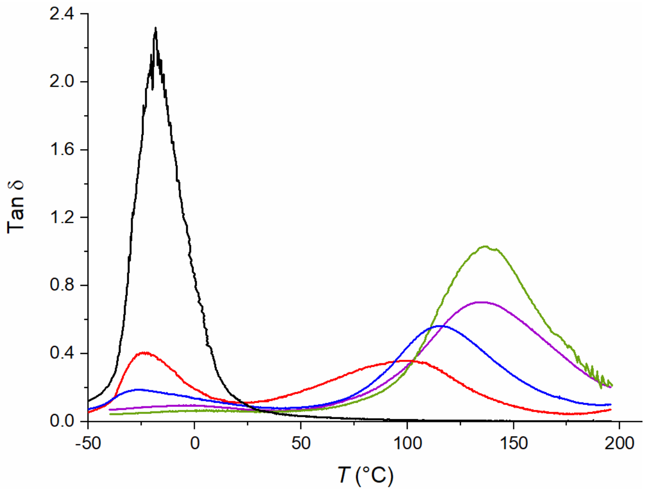

Supplementary Figure 10. 5% (red), 10% (blue), 20% (purple), and 30% (green) C4ASA-films' tan δ as a function of temperature. Each film contains 1% BudMA.

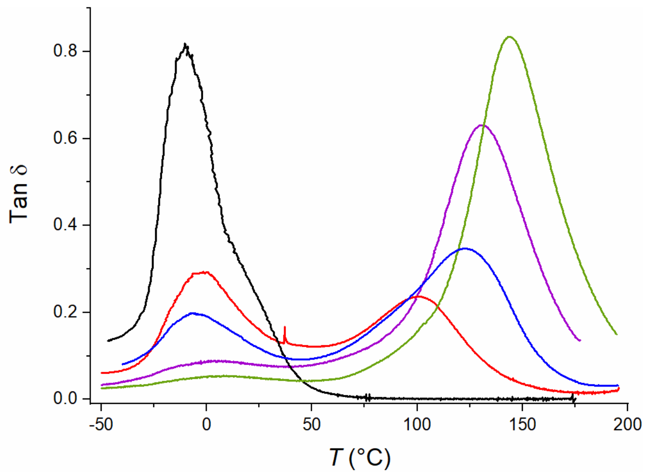

Supplementary Figure 11. 0% (black), 5% (red), 10% (blue), 20% (purple), and 30% (green) C4ASA-films' tan δ as a function of temperature. Each film contains 5% BudMA.

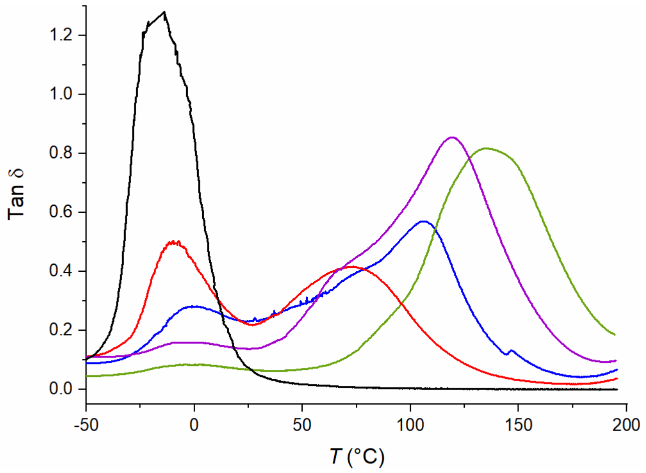

Supplementary Figure 12. 0% (black), 5% (red), 10% (blue), 20% (purple), and 30% (green) C6ASA-films' tan δ as a function of temperature. Each film contains 2% BudMA.

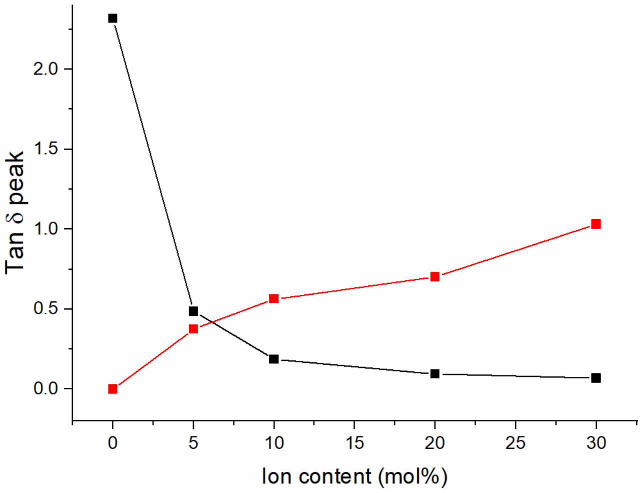

Supplementary Figure 13. The tan δ peak heights of C4ASA-films as a function of ion content. The films are crosslinked with 1% BudMA. The matrix peak heights are given in black, and the cluster peaks are given in red.

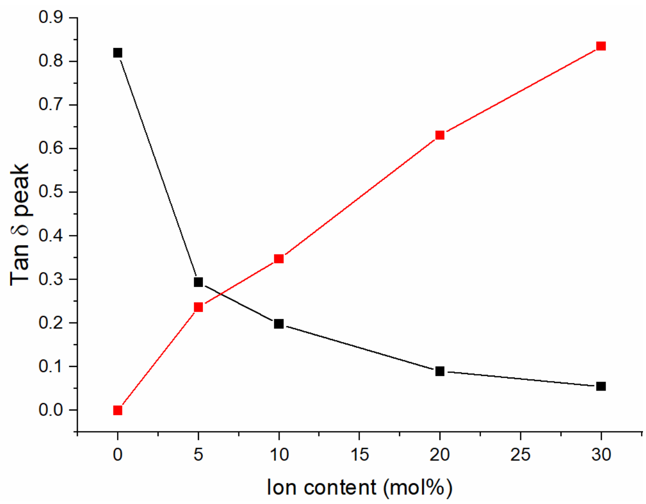

Supplementary Figure 14. The tan δ peak heights of C4ASA-films as a function of ion content. The films are crosslinked with 5% BudMA. The matrix peak heights are given in black, and the cluster peaks are given in red.

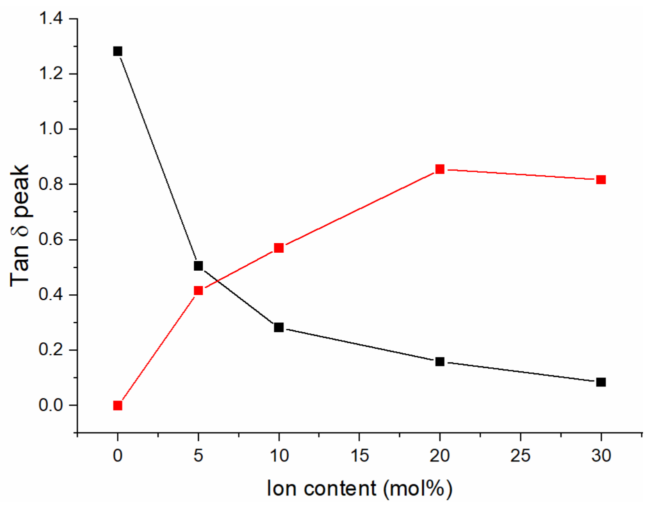

Supplementary Figure 15. The tan δ peak heights of C6ASA-films as a function of ion content. The films are crosslinked with 2% BudMA. The matrix peak heights are given in black, and the cluster peaks are given in red.

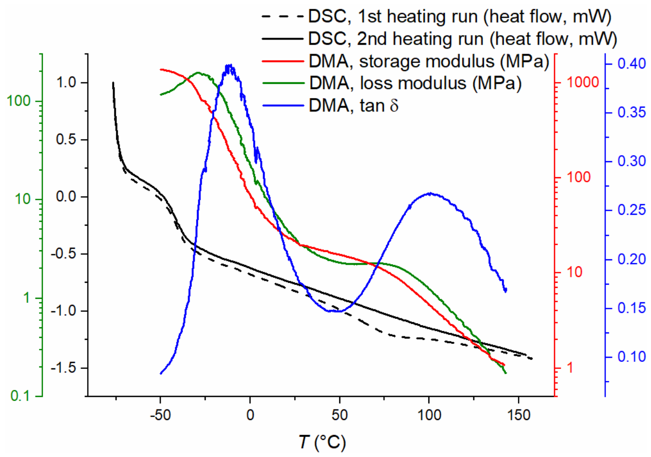

Supplementary Figure 16. Comparison of the DMA and DSC data of a film with 2% BudMA and 5% C4ASA.

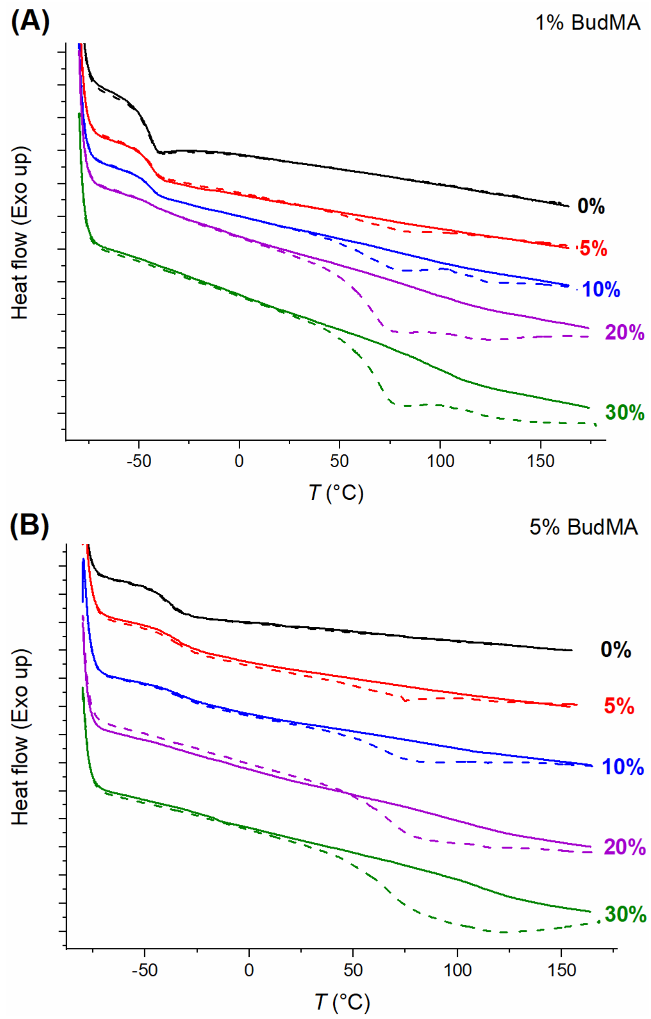

Supplementary Figure 17. The DSC traces of C4ASA-films with (A) 1% BudMA and (B) 5% BudMA. The films contain 0% (black), 5% (red), 10% (blue), 20% (purple), or 30% (green) C4ASA. The first heating runs are depicted with dashed lines and the second heating runs with solid lines. The traces are shifted along the y-axis for comparison.

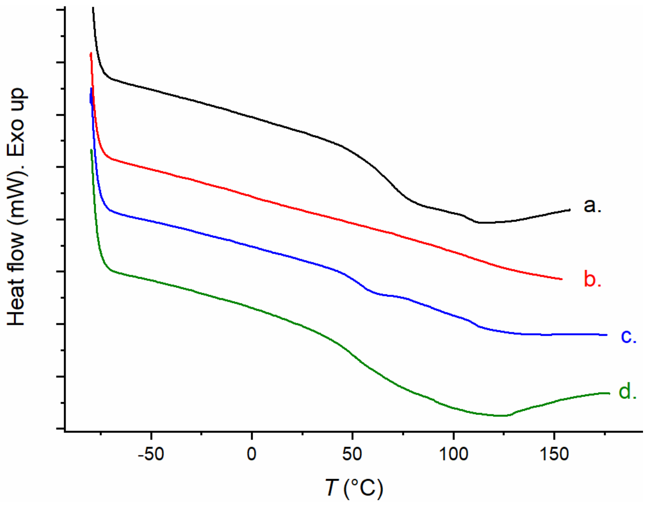

Supplementary Figure 18. DSC traces of a film with 2% BudMA and 30% C4ASA. Same sample was heated four times; a. the first heating, b. 5 minutes after the first heating-cooling cycle, c. after keeping the sample at room temperature for three days, and d. 91 days after the previous heating run. The traces are shifted along the y-axis for comparison.

4 The tensile properties of the materials

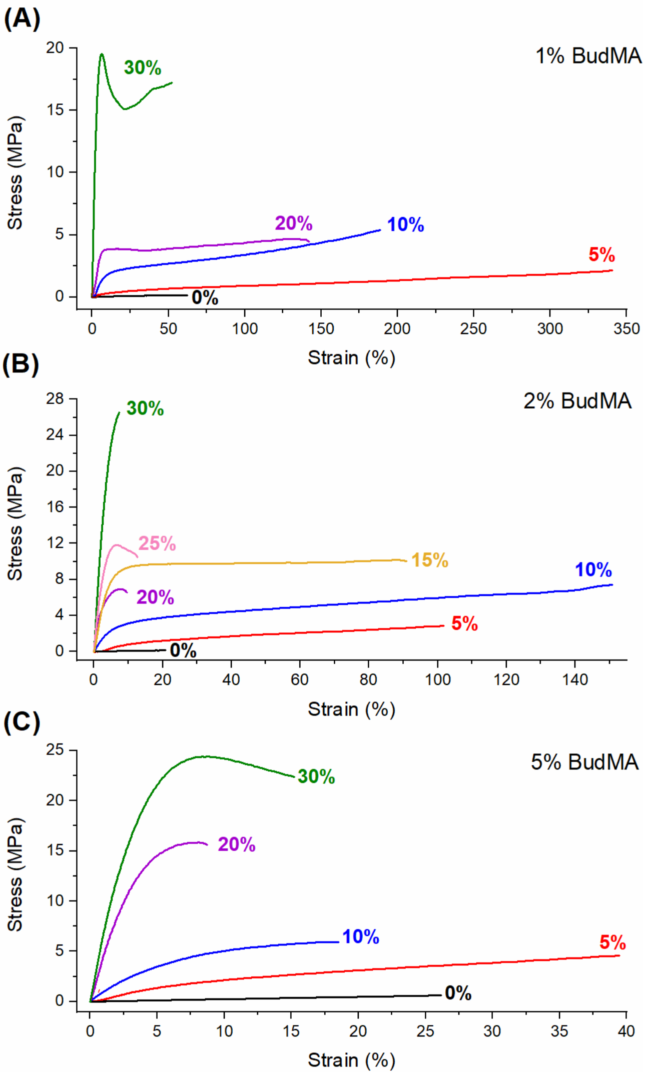

Supplementary Figure 19. The stress-strain curves of the C4ASA-containing films crosslinked with (A) 1% BudMA, (B) 2% BudMA, and (C) 5% BudMA. The stress-strain curves are labeled with their respective ion contents.

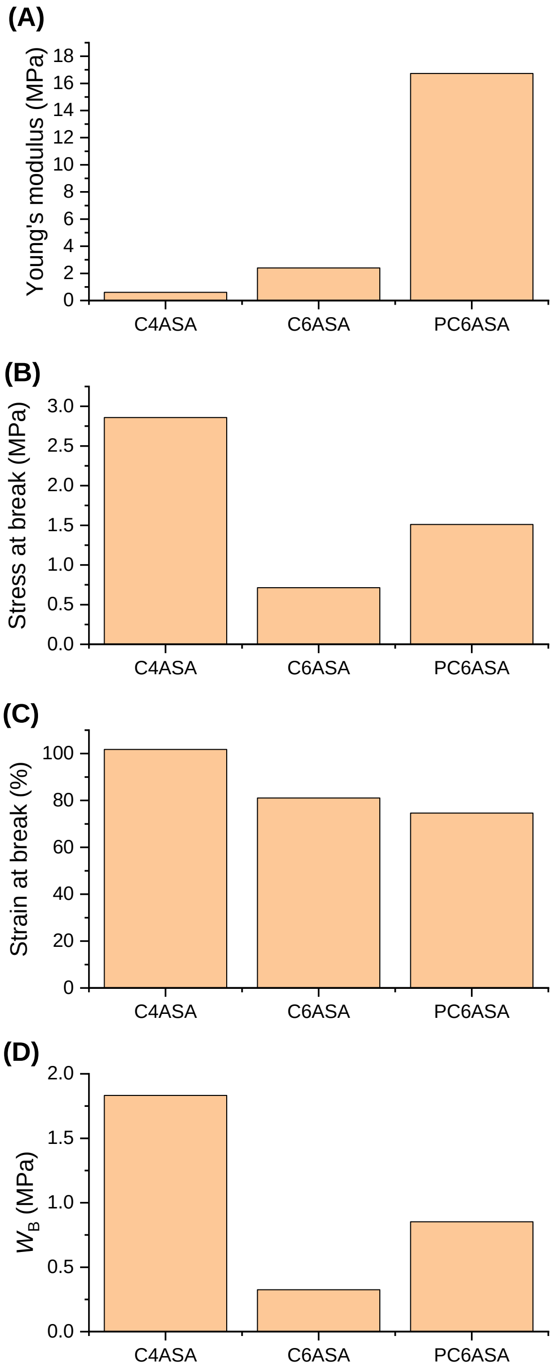

Supplementary Figure 20. The Young's moduli (A), stresses at break (B), strains at break (C), and fracture energies (D) of films with 5% of C4ASA, C6ASA, or PC6ASA. Each film contains 2% BudMA.
